# IgG-like bispecific antibodies with potent and synergistic neutralization against circulating SARS-CoV-2 variants of concern

**DOI:** 10.1038/s41467-022-33030-4

**Published:** 2022-10-03

**Authors:** Matthew R. Chang, Luke Tomasovic, Natalia A. Kuzmina, Adam J. Ronk, Patrick O. Byrne, Rebecca Johnson, Nadia Storm, Eduardo Olmedillas, Yixuan J. Hou, Alexandra Schäfer, Sarah R. Leist, Longping V. Tse, Hanzhong Ke, Christian Coherd, Katrina Nguyen, Maliwan Kamkaew, Anna Honko, Quan Zhu, Galit Alter, Erica Ollmann Saphire, Jason S. McLellan, Anthony Griffiths, Ralph S. Baric, Alexander Bukreyev, Wayne A. Marasco

**Affiliations:** 1 Department of Cancer Immunology & Virology, Dana-Farber Cancer Institute, Boston, MA 02215 USA; 2 Department of Pathology, University of Texas Medical Branch, Galveston, TX 77555 USA; 3 Galveston National Laboratory, Galveston, TX 77555 USA; 4 Department of Microbiology and Immunology, University of Texas Medical Branch, Galveston, TX 77555 USA; 5 Department of Molecular Biosciences, University of Texas, Austin, TX 78712 USA; 6 Department of Microbiology and National Emerging Infectious Diseases Laboratories, Boston University, School of Medicine, Boston, MA 02118 USA; 7 La Jolla Institute for Immunology, La Jolla, CA 92037 USA; 8 Department of Epidemiology, University of North Carolina at Chapel Hill, Chapel Hill, NC 27599 USA; 9 Department of Medicine, Harvard Medical School, Boston, MA 02115 USA; 10 Ragon Institute of MGH, MIT and Harvard, Cambridge, MA 02139 USA

**Keywords:** Infectious diseases, Antibody therapy, Cryoelectron microscopy, SARS-CoV-2

## Abstract

Monoclonal antibodies are a promising approach to treat COVID-19, however the emergence of SARS-CoV-2 variants has challenged the efficacy and future of these therapies. Antibody cocktails are being employed to mitigate these challenges, but neutralization escape remains a major challenge and alternative strategies are needed. Here we present two anti-SARS-CoV-2 spike binding antibodies, one Class 1 and one Class 4, selected from our non-immune human single-chain variable fragment (scFv) phage library, that are engineered into four, fully-human IgG-like bispecific antibodies (BsAb). Prophylaxis of hACE2 mice and post-infection treatment of golden hamsters demonstrates the efficacy of the monospecific antibodies against the original Wuhan strain, while promising in vitro results with the BsAbs demonstrate enhanced binding and distinct synergistic effects on neutralizing activity against circulating variants of concern. In particular, one BsAb engineered in a tandem scFv-Fc configuration shows synergistic neutralization activity against several variants of concern including B.1.617.2. This work provides evidence that synergistic neutralization can be achieved using a BsAb scaffold, and serves as a foundation for the future development of broadly reactive BsAbs against emerging variants of concern.

## Abstract (web summary)

COVID-19 can be treated with monoclonal antibodies against SARS-CoV-2, but emerging new variants might show resistance towards existing therapy. Here authors show that anti-SARS-CoV-2 spike human single-chain antibody fragments could gain neutralizing activity against variants of concern upon engineering into a human bispecific antibody.

## Introduction

The spike proteins of SARS-CoV-2 are considered to be prime antibody targets as they are readily accessible on the viral surface and have an essential role in viral attachment and infection of host cells. Neutralizing anti-spike antibodies can block the virus’ ability to infect new cells and in doing so put pressure on the virus to undergo neutralization escape. Thus, the occurrence of COVID-19 variants is an expected development and is driving the evolving pandemic with continual loss of life, placing added burden on our healthcare system and uncertainty around the U.S. and global recovery. While not commonly used at the beginning of the pandemic, monoclonal antibody (mAb) therapies are gaining in popularity and numerous infusion sites have been deployed around the country^1^. These therapies typically provide protection from viral infection by inhibiting viral binding and entry into target cells, and unlike a vaccine, are not dependent on initiating an endogenous response from the host and can be administered post-infection. While traditional antibody (Ab) therapies were designed using a single neutralizing antibody (nAb), current anti-viral therapies often combine two nAbs as a cocktail that is delivered as a single therapeutic dose.

The rationale for this cocktail Ab approach is clearly illustrated when comparing the efficacy and success of Lilly’s monotherapy bamlanivimab (LY-CoV555) and Regeneron’s combination therapy of casirivimab (REGN10933) with imdevimab (REGN10987), both of which were granted Emergency Use Authorization (EUA) in late 2020. As a monotherapy, bamlanivimab and casirivimab display weakness against B.1.351 (Beta); however, when casirivimab is administered in combination with imdevimab, high therapeutic efficacy is maintained^2^. As bamlanivimab was originally administered as a monotherapy, the sensitivity to the B.1.351 strain lead to an increased risk of treatment failure and therefore the FDA revoked bamlanivimab’s monotherapy EUA in April 2021^2^. To counter this, Lilly developed a second mAb (etesevimab, LY-CoV016) to be used in combination with bamlanivimab and was able to regain EUA approval for the combination therapy. In addition to the therapeutic benefits of combination therapies, AstraZeneca recently announced preliminary phase III results demonstrating that prophylactic administration of tixagevimab (AZD8895) with cilgavimab (AZD1061) significantly reduced the incidence of symptomatic COVID-19.

Bispecific antibodies (BsAb) combine the antigen binding domains from two mAbs onto one framework, maintaining the advantage over the emergence of escape mutants without the need to produce two separate mAbs for cocktail therapy. BsAbs are classified into one of two categories, IgG-like or non-IgG like. IgG-like BsAbs contain an Fc domain allowing them to engage effector functions and constructs range from the original asymmetric knob-in-holes, to multivalent IgG-scFv fusions, to the highly optimized cross-over dual variable (CODV)-Igs^3–6^. Non-IgG like BsAbs include diabodies and dual-affinity re-targeting antibodies (DART) and are built using linked variable regions without Fc domains^7,8^. Smaller and more agile, these non-IgG like BsAbs provide rapid biodistribution and organ penetration. However, their size and lack of an Fc region allows them to be promptly cleared by the kidneys^9^. Although a few BsAbs are currently approved by the FDA for treatment of non-infectious diseases and anti-influenza and Ebola BsAbs have been reported to exhibit increased potency compared to the parental mAbs, only two BsAbs for the treatment of HIV based on trans-recognition of T cells and HIV are in clinical trials and no anti-viral BsAbs have received regulatory approval^10–16^.

Here we report on the design and activity of four anti-SARS-CoV-2 BsAbs. One tetravalent BsAb design using a Class 4 and 1 tandem pair results in potent and synergistic neutralization against Wuhan strain and numerous variants of concern. This strategy of tethering two Abs targeting different epitopes on the spike may provide a sufficient gain of affinity to mitigate loss of binding and neutralization activity. Our data provides evidence for the superior therapeutic efficacy of the tandem scFv-Fc Ab 2-7/Ab 12 and supports the foundational role of anti-SARS-CoV-2 BsAbs for the next generation of prophylactic and therapeutic agents to combat emerging COVID-19 variants.

## Results

### Discovery and in vitro characterization of Ab 12 and 2-7

**Fig. 1:**
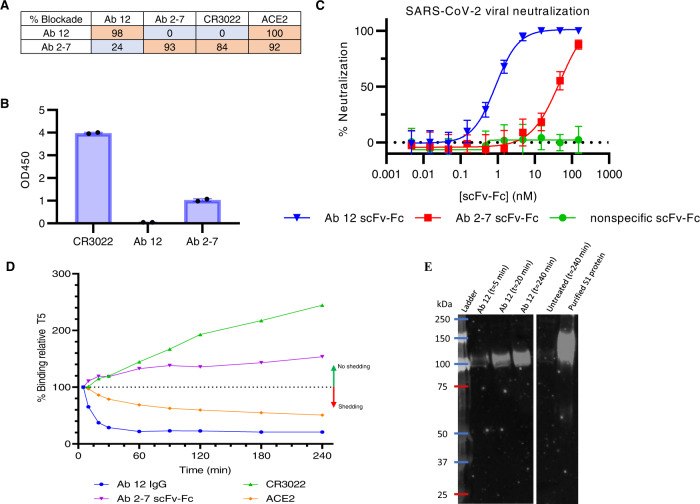
Biochemical characterization of Ab 12 and Ab 2-7 scFv-Fcs. **A** Competition matrix showing percent blockade of CR3022 and ACE2 binding to SARS-CoV-2 RBD. **B** Antibody binding at 2 ug/ml was measured by ELISA against Tor2 SARS-CoV spike (Toronto, 2003). *n* = 2 biologically independent samples and the mean is presented. **C** PRNT neutralization assays performed with SARS-CoV-2 (isolate USA‐WA1/2020) demonstrate that Ab 12 is the more potent of the two scFv-Fcs. Samples were tested as 3 biologically independent samples with 3 technical replicates per sample and the data is presented as the mean with standard deviation. **D** FACS based measurement of spike shedding induced by binding of Ab 12, Ab 2-7, CR3022 IgG, and solACE2 at 37 °C. A decrease in median fluorescence indicates an increase in spike shedding whereas an increase in fluorescence indicates minimal shedding is observed (*n* = 2 biologically independent samples with the mean represented). **E** Western blot detecting shed S1 in supernatant from Ab 12 IgG spike shedding experiment confirming decreasing fluorescence is at least partially a result of shedding. Experiment was repeated once and representative data is presented. Source data are provided as a Source Data file.

The Marasco Lab previously generated a 27-billion-member naïve phage library via random assembly of heavy and light chain variable genes isolated from peripheral blood of 57 healthy donors. Using this library, a phage panning campaign was completed using SARS-CoV-2 S1 and the receptor binding domain (RBD), leading to the discovery of anti-SARS-CoV-2 RBD antibodies Ab 12 and Ab 2-7. Kinetic parameters for the monovalent fragments of Ab 12 (Fab) and Ab 2-7 (scFv) were measured via biolayer interferometry (BLI) and both had nanomolar affinity to the SARS-CoV-2 RBD (Supplementary Fig. 1A, B). Initial epitope binning was performed with the bivalent scFv-Fcs via competition with ACE2 and CR3022, as these two proteins have been demonstrated to bind opposite ends of the RBD^17^. This screening revealed that while both Ab 12 and Ab 2-7 block ACE2 binding, only Ab 2-7 inhibited CR3022 binding to the RBD (Fig. 1A). In addition to competing with both ACE2 and CR3022, Ab 2-7 binds the Tor2 spike protein, a SARS-CoV strain isolated in Toronto circa 2003, indicating that it likely binds the more conserved RBD core (Fig. 1B)^18^.

Ab 12 and Ab 2-7 were next tested for SARS-CoV-2 neutralization via plaque reduction neutralization tests (PRNT) and recombinant nLuc viruses. Both Abs neutralized SARS-CoV-2, although Ab 12 scFv-Fc was significantly more potent with an IC_50_ of 0.86 nM compared to 47.36 nM for Ab 2-7 in PRNT assays (Fig. 1C). While blockade of RBD-ACE2 interactions is the most commonly observed mechanism of antibody neutralization, other mechanisms have also been identified, including destabilization of the pre-fusion spike trimer, premature shedding of the S1 domain and conversion to the post-fusion conformation, and restricting access to the post-fusion conformational state^19–21^. Among these, destabilization of the spike trimer and premature shedding of the S1 domain are preferred methods of neutralization, as both of these are irreversible and permanently disable the spike protein from binding ACE2. Conversely, blockade of ACE2 binding and locking the spike in the pre-fusion conformation are transient mechanisms that are reversed upon antibody disassociation^22^. Having demonstrated that our antibodies block ACE2 binding to the RBD, we looked to determine if our antibodies are able to neutralize through one of the additional mechanisms using a spike shedding experiment similar to that developed by Wec et al.^23^. 293T cells were transduced to constitutively express the SARS-CoV-2 spike on the cell surface and as shown in Fig. 1D, treatment of these cells with Ab 12 leads to decreased levels of surface-bound antibody over time, suggesting that Ab 12 treatment leads to the loss of surface spike molecules. Though antibody-induced endocytosis potentially contributes to the decreased cell surface antibody detected, a time-dependent increase in the concentration of S1 in the supernatant following treatment with Ab 12 is observed (Fig. 1E), providing evidence that Ab 12 triggers the premature and irreversible shedding of the S1 domain. As these two antibodies have been demonstrated to target non-overlapping epitopes and utilize varying mechanisms of neutralization, Ab 12 and Ab 2-7 were chosen for further evaluation and engineering.

### Structural analysis of Ab 12 and Ab 2-7

**Fig. 2:**
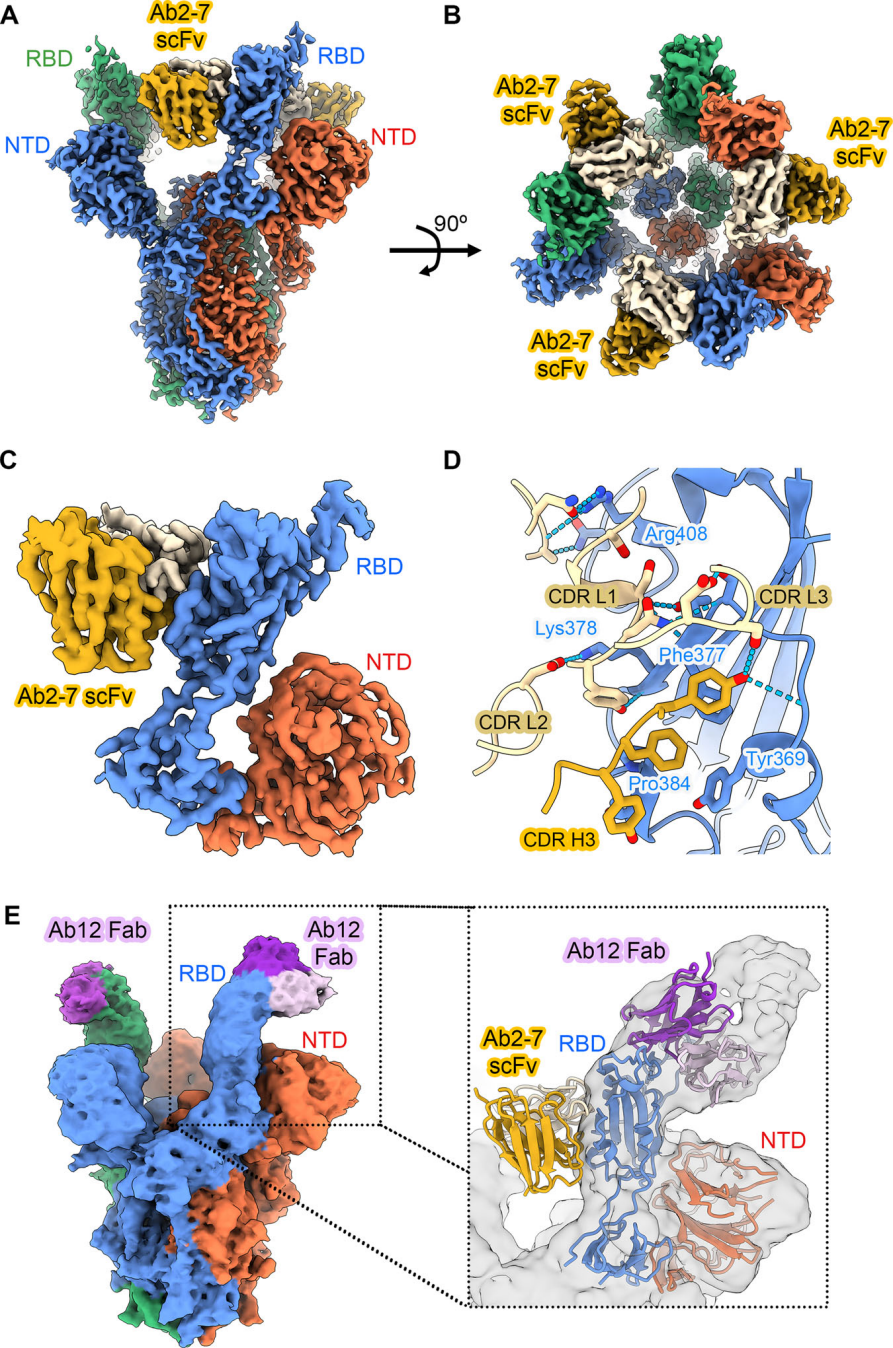
High resolution cryo-EM structure of Ab 2-7 scFv and Ab 12 Fab complexed SARS-CoV-2 S D614G. **A** Side and (**B**) top views of a composite 3D cryo-EM reconstruction of Ab 2-7 scFvs bound to the receptor binding domains of SARS-CoV-2 spike ectodomains at 3.0 Å resolution. Individual spike protein protomers are colored red, blue and green. The heavy chain of Ab 2-7 scFv is colored yellow, the light chain is colored tan. **C** Locally refined 3D cryo-EM reconstruction of antibody binding interface. **D** Atomic model of the binding interface between Ab 2-7 scFv and the spike RBD. **E** Side view of 3D EM reconstruction of Ab 12 Fab (purple) bound to the RBD of SARS-CoV-2 spike ectodomain. Overlay of the Ab 2-7 and Ab 12 structures demonstrates that the two antibodies target spatially discrete, non-overlapping epitopes (insert, with the Ab 12 cryo-EM reconstruction shown in gray).

To examine the mode of binding of Ab 2-7 to SARS-CoV-2 spike, we initiated structural studies by electron microscopy (EM). We obtained two high-resolution 3D cryo-EM reconstructions of Ab 2-7 scFv complexed with SARS-CoV-2 S D614G: one with two scFvs bound to a spike trimer, and the other with three scFvs bound (3.4 Å and 3.0 Å respectively, Supplementary Fig. 2, Fig. 2A, B). We also determined a structure of this variant in the absence of any ligands to facilitate comparison with the scFv-bound structures (3.0 Å, Supplementary Fig. 2). Local refinement of a masked region comprising the spike RBD, NTD, and Ab 2-7 scFv improved the quality of the 3D reconstruction for the 3-scFv-bound structure (Supplementary Fig. 3, Fig. 2C), allowing us to conclude that Ab 2-7 binds to the non-receptor binding motif (RBM) core of the RBD using a mix of heavy and light chain interactions from CDR H3 and L1-L3 as well as two residues from the framework region of the light chain. The interface features a dozen hydrogen bonds, five π-π stacking interactions, and 829 Å of buried surface area (Fig. 2D) with the π-π interactions clustered around three residues in the non-RBM core of the spike RBD: Y369, F377, and P384.

**Fig. 3:**
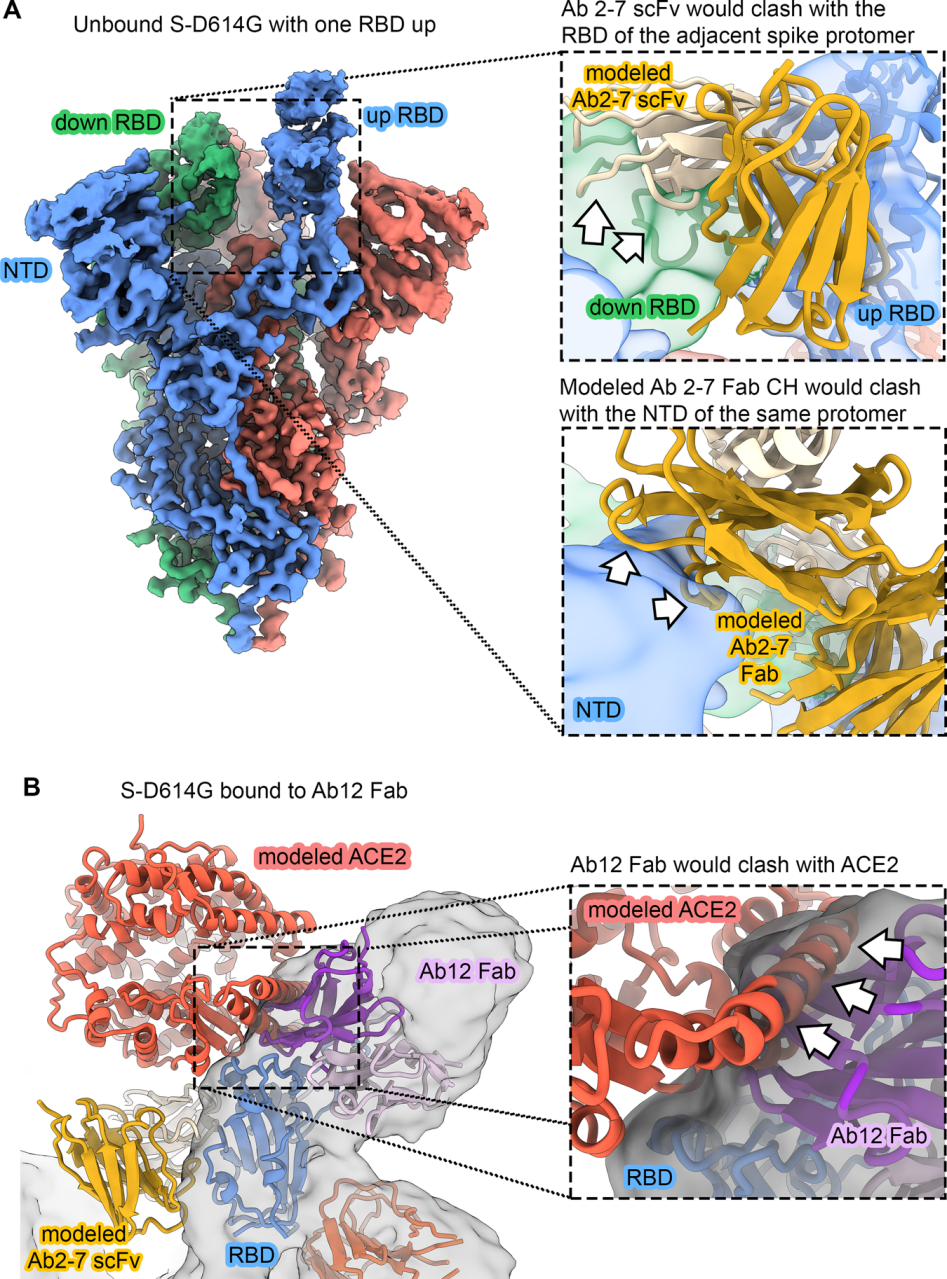
Ab 2-7 can only bind as an scFv and Ab 12 competes with ACE2. **A** Cryo-EM reconstruction of unbound S-D614G with one RBD in the up conformation (3.0 Å). Coloring is the same as in Fig. 2. The inset at the top right shows that Ab 2-7 would clash with the “down” RBD of the neighboring spike protomer (green cryo-EM reconstruction, filtered to 6 Å). The inset below shows Ab 2-7 modeled as a Fab, wherein the CH region would clash with the spike NTD (blue cryo-EM reconstruction, filtered to 6 Å). Clashes are indicated as white arrows. **B** Overlay of ACE2 bound to the RBD confirms that Ab 12 directly competes for the receptor binding motif (RBM). Ab 2-7 binds the conserved RBD core and does not directly compete with ACE2. Clashes are indicated as white arrows.

Comparison of the three structures (zero, two and three Ab 2-7 scFvs bound) sheds light on the mechanism of Ab 2-7 scFv binding to the spike trimer. The structure of spike trimer bound to two Ab 2-7 scFv molecules shows the third unbound RBD in the up conformation. Superposition with the apo-spike structure reveals that Ab 2-7 scFv would clash with the RBD of the neighboring spike protomer if it were in the down-conformation and therefore cannot fully engage its binding epitope until at least two RBDs are in the up conformation (Fig. 3A top inset). Once bound, Ab 2-7 scFv prevents the RBD from accessing the down conformation. Furthermore, superposition of Fab constant regions (CH1/CL) onto the Ab 2-7 scFv structure indicates that the CH1/CL domains would clash with the NTD of the neighboring protomer (Fig. 3A bottom inset). Ab 2-7 scFv thus binds to the spike in a manner that would be inaccessible to its Fab form.

Cryo-EM structures were also solved for the Ab 12 Fab in complex with the ectodomain of the SARS-CoV-2 spike at 3.6 Å resolution. The 3D classification of the particles showed the presence of spike trimers with either one or two Ab 12 Fabs bound at once. Similar to Ab 2-7, Ab 12 also targets the RBD in the up-configuration (Fig. 2E), however Ab 12 targets the RBM, recognizing an epitope centered over the RBD saddle region, spanning the RBD edge to the side of the RBD ridge (Fig. 2E) and overlay with ACE2 confirms that this antibody directly inhibits ACE2 binding (Fig. 3B)^24,25^. Additionally, the cryo-EM experiments demonstrated that Ab 12 binding mimicked ACE2-mediated triggering of the conformational change to the post-fusion state, as indicated by the presence of a significant number of destabilized spike monomers in solution, further supporting the mechanistic studies in Fig. 1D. As an RBM targeting Ab that binds the peak of the RBD in the up conformation, Ab 12 can be classified as a Class 1 or RBD-2 antibody^26,27^. Conversely, Ab 2-7 targets an epitope on the conserved RBD core, partially overlapping with the broadly-neutralizing Ab S2X259^28^. This epitope is only accessible in the two RBD up conformation, competes with ACE2, and results in moderate viral neutralization, indicating that this is an RBD-7a (Class 4) Ab^26,27^.

### In vivo neutralization in Syrian golden hamsters

**Fig. 4:**
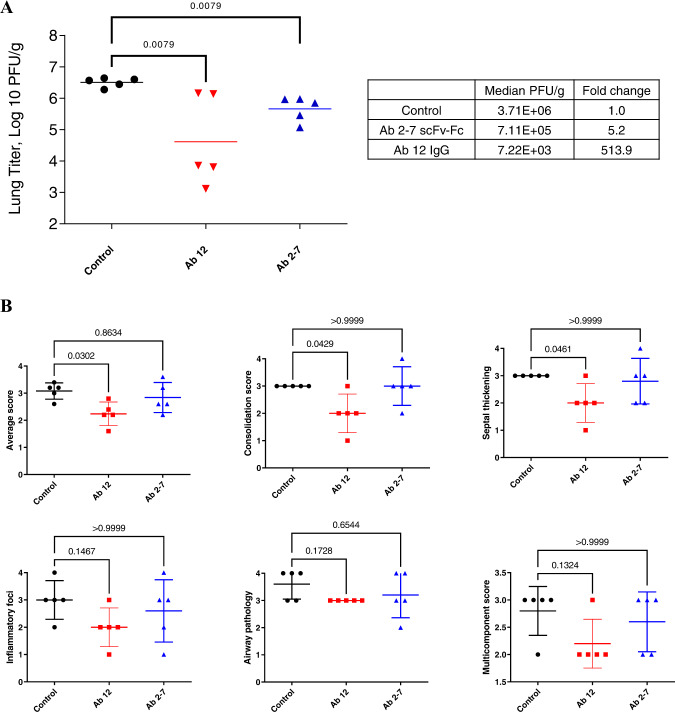
Therapeutic efficacy of Ab 12 IgG and Ab 2-7 scFv-Fc in Syrian golden hamster model. **A** Therapeutic treatment of Syrian golden hamsters post-infection with Ab 12 IgG or Ab 2-7 scFv-Fc leads to a 513.9- and 5.2-fold reduction of viral loads, respectively, compared to control (PBS) treated animals **B** Pathology scores for animals treated with PBS, Ab 12 IgG, or Ab 2-7 scFv-Fc. Significant reduction in overall, consolidation, and septal thickening scores were seen for Ab 12 treatment. Scores were determined based on the criteria in Supplementary Table 2. Data points represent individual mice with geometric mean (**A**) or mean ± standard deviation in (**B**). Statistical analysis was performed using Mann–Whitney test in (**A**) and Kruskall–Wallis test with Dunn’s post-hoc correction in (**B**). Source data are provided as a Source Data file.

The in vivo biological activity of these mAbs was next tested in Syrian golden hamsters, a model for severe COVID-19^29,30^. Consistent with in vitro data, Ab 12 treatment 1 day post intranasal viral challenge resulted in a significant decrease (>500 fold) in the lung viral titers compared to untreated animals (Fig. 4A). Pathological analysis of the lung tissues showed that compared to control-treated animals, Ab 12 treatment leads to marked reduction in consolidation, smaller foci of infiltration, and minimal perivascular cuffing (Fig. 4B, S6). In contrast, Ab 2-7 scFv-Fc treatment resulted in only a 5-fold decrease in titer compared to control treated animals (Fig. 4A). Pathologic scoring demonstrated that Ab 2-7 treatment did not significantly reduce consolidation or inflammatory infiltration although a modest decrease in the number of inflammatory cells in the airways and in severity of the perivascular cuffing was observed (Fig. 4B, S6). Thus, while Ab 12 IgG showed therapeutic benefit when used alone, only modest anti-viral effects were seen with Ab 2-7 scFv-Fc.

### Development and characterization of bispecific antibodies

**Fig. 5:**
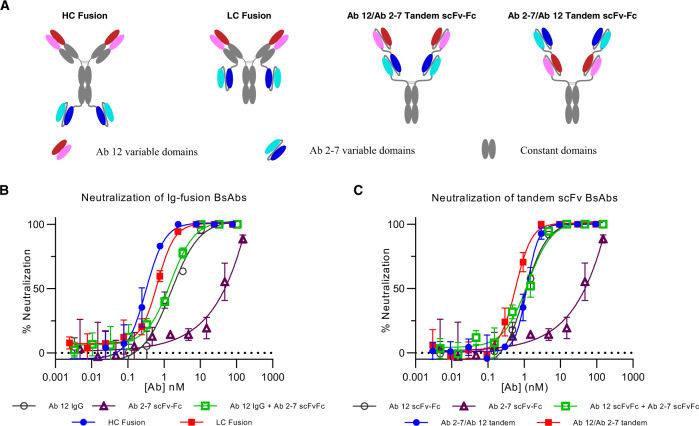
Design and in vitro characterization of anti-SARS-CoV-2 BsAbs. **A** Design of the four anti-SARS-CoV-2 BsAbs. Constant regions are colored in gray, Ab 12 binding domains are red/pink, and Ab 2-7 binding domains are blue/cyan. In vitro neutralization was performed with the IgG fusion BsAbs (**B**) and tandem scFv-Fcs (**C**). Samples were tested with *n* = 3 biologically independent samples and data are presented as mean ± standard deviation. Source data are provided as a Source Data file.

Coronaviruses are positive-sense, single-stranded RNA viruses that are characterized by high mutation rates. The mutagenic capability of a virus depends upon several factors, including the fidelity of viral enzymes that replicate nucleic acids and these mutations drive viral evolution and genome variability^31–33^. While many naturally occurring mutations do not have a significant impact on viral fitness, some mutations can have a detrimental effect leading to viral extinction, and other more rare mutations promote viral evolution and escape from anti-viral therapies. This is demonstrated by the diversity of variants circulating today and the primary challenge to mAb therapies are mutations that arise in the spike protein within the mAb binding site as a result of inaccuracies during viral replication combined with immune pressure^26,34–36^. To counter the emergence of these mutations, we used Ab 12 and Ab 2-7 to develop a series of BsAbs targeting their spatially discrete epitopes on the RBD, with the intent of raising the threshold required for SARS-CoV-2 variants of concern to undergo neutralization escape. Four BsAbs were developed, two are IgG fusions, using the more potent Ab 12 as the IgG scaffold with an Ab 2-7 scFv fused to the C-terminus of either the heavy or light chain constant regions via a flexible linker. Two additional BsAbs were developed using both orientations of a tandem scFv arrangement, fused to an IgG1 Fc via an CD8 hinge domain (Fig. 5A, S7).

As with the monospecific Abs, in vitro neutralization activity of these BsAbs was tested in PRNT assays. While the IgG fusion BsAbs show a shift to the left in the dose–response curve compared to the parental mixture and individual antibodies, the tandem scFv BsAbs display a more subtle shift in efficacy (Fig. 5B, C). To further investigate the prophylactic potential of the BsAbs, we tested the constructs in vitro and in vivo using a mouse-adapted SARS-CoV-2 virus^37^. The RBD of the mouse-adapted strain has two mutations that allow for binding to mouse ACE2 and these mutations lie on the edge of the Ab 12 binding site (Supplementary Fig. 8A). Against this mouse-adapted strain, Ab 12 IgG showed an approximately one log loss of neutralization activity compared to Ab 12 scFv-Fc but no difference in neutralization activity between these Ab 12 formats was seen against the WT Wuhan strain (Supplementary Fig. 8B). In vivo against the mouse-adapted strain, the HC Fusion displayed the greatest potency among the BsAbs and was superior to the parental Ab 12 IgG monotherapy, however the two Ab cocktail therapies displayed the greatest potency overall (Supplementary Fig. 9A). To determine if the RBD mutations Q498Y and P499T in the mouse-adapted stain negatively affected the efficacy of our Abs, we chose one of the most potent overall treatments (Ab 12 + Ab 2-7 scFv-Fcs) and the most potent BsAb (HC Fusion) for testing in a transgenic humanACE2 mice using WT SARS-CoV-2 virus. Supplementary Fig. 9B demonstrates that prophylactic treatment with either the scFv-Fc combination or HC Fusion leads to viral titers below the limit of detection, except for one mouse treated at the lowest concentration of BsAb, confirming that the results seen in the mouse-adapted virus for these two treatments were likely skewed by the RBD mutations.

#### Binding analysis of mono- and bi- specific antibodies to circulating SARS-CoV-2 variants

The D614G spike mutation increases the stability of the spike protein and favors the RBD to maintain the up conformation, leading to increased ACE2 accessibility and thereby infectivity, allowing it to briefly become the predominant strain circulating worldwide^38–40^. Using this mutation as a template, the first variant of concern, B.1.1.7 (Alpha), soon emerged and as of December 2021, four additional variants of concern have been identified, B.1.351 (Beta), P.1 (Gamma), B.1.617.2 (Delta), and B.1.1.529 (Omicron), each containing a unique combination of spike mutations resulting in varying levels serum and monoclonal antibody evasion^41–43^.

**Fig. 6:**
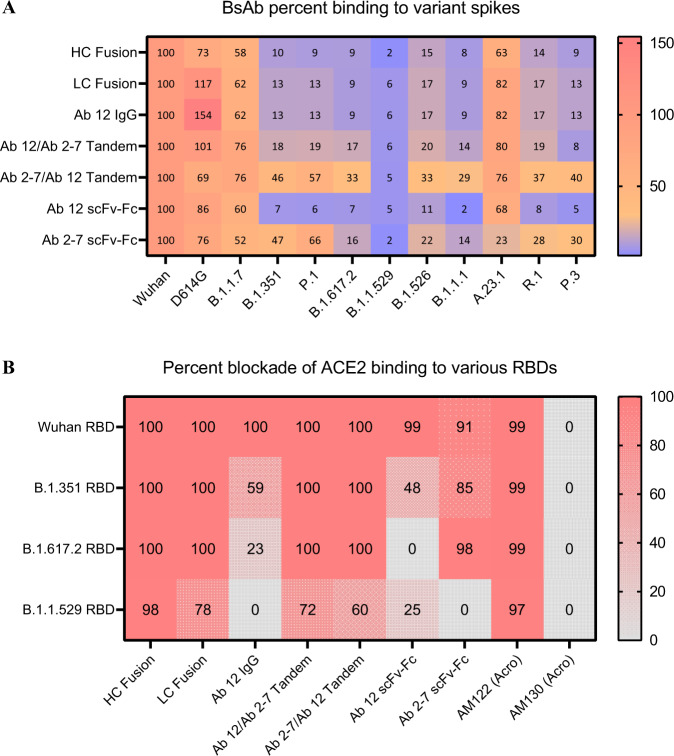
BLI binding and competition analysis for mono- and bispecific constructs with various variant spike/RBD proteins. **A** AHC sensors were loaded with anti-SARS-CoV-2 mono- and bispecific Abs and then dipped into wells containing different Spike variants. Amplitude of the binding signal for each variant was normalized to that Ab format’s binding of the Wuhan spike. Though the parental Abs, IgG fusions, and Ab 12/Ab 2-7 tandem BsAbs exhibit marginal affinity to the variants tested, Ab 2-7/Ab 12 constantly binds at a greater level than either of the parental Abs, indicating a synergistic binding pattern. **B** Competition with ACE2 for binding to Wuhan, B.1.351, B.1.617.2, and B.1.1.529 RBDs was performed with biotinylated RBD proteins and soluble ACE2. Though our BsAbs weakly bind mutant spikes at low concentration, at high concentrations they are able to effectively block ACE2 binding to the RBD. Two chimeric antibodies from Acro Biosystems were used as experimental controls, as both are cross-reactive but only AM122 has been demonstrated to compete with ACE2 binding. The percent binding experiment was performed in triplicate and mean values are shown. Competition was performed as a single experiment. Source data are provided as a Source Data file.

To determine the effect of these mutations on the affinity of our mono- and bi- specific Abs, binding experiments were conducted using a panel of SARS-CoV-2 variants. Fig. 6A shows the level of binding to soluble forms of the spike variants, normalized to that of wildtype spike. While not sufficient to escape the polyclonal response in vaccinated and convalescent individuals, the RBD mutation (N501Y) in B.1.1.7 has demonstrated the ability to reduce the potency of neutralizing antibodies targeting epitopes that include N501, however neutralizing antibodies targeting adjacent epitopes remain potent^44^. The D614G mutation has been found to increase stability of the D614G spike, which allows for increased binding of nearly all constructs tested. A moderate loss of binding was observed for all antibody constructs for the B.1.1.7 spike compared to the WT spike, suggesting that the epitopes targeted by Abs 12 and 2-7 are partially affected by the perturbations generated by the N501Y mutation seen in B.1.1.7. Conversely, the B.1.351, P.1, and B.1.1.529 lineages contain three RBD mutations (K417, E484, and N501) which have been shown to evade plasma neutralization and reduce the affinity of both bamlanivimab (Class 2) and casirivimab (Class 1)^42,45–47^. Ab 12 (Class 1) is no exception to this and displays a circa 90% drop in binding capacity for these variant spikes (Fig. 6A). Though Ab 2-7 is partially protected from these mutations in B.1.351 and P.1 with only a 50% decrease in binding, it is unable to overcome the vast number of mutations seen in the B.1.1.529 spike (Fig. 6A).

Surprisingly, in an ACE2 competition assay using variant RBDs, all of our BsAb display increased ACE2 blockade compared to the parental mAbs, with the most impressive improvement seen with the HC Fusion and B.1.1.529 RBD as almost all ACE2 binding is blocked (Fig. 6B). Though initially contrary to the results in Fig. 6A, the increase in binding of the BsAbs and ACE2 blockade potential likely arises from the increased avidity seen with the tetravalent bispecifics at saturating conditions compared to sub-saturating conditions used in the binding assay. This is further supported by the fact that even under saturating conditions, bivalent Ab 12 struggles to bind the variant RBDs at high enough levels to efficiently block ACE2 binding, and both Ab 12 and Ab 2-7 demonstrate minimal blockade of the B.1.1.529 RBD (Fig. 6B).

Overall, our antibodies exhibited relatively weak binding to the majority of the variants tested, with Ab 12 and the IgG fusions exhibiting near complete loss of binding. The exception to this is the Ab 2-7/Ab 12 tandem scFv-Fc construct that demonstrated only moderate loss of binding to all variants except for B.1.1.529 (Fig. 6A). This suggests that leading with the Ab 2-7 scFv in the tandem BsAb provides better access to both antibody binding sites compared to the reverse tandem orientation, enabling enhanced binding. Combined with the lack of binding seen in Fig. 6A across all mono- and BsAbs, the decision was made not to test B.1.1.529 neutralization in vitro.

### In vitro neutralization of circulating SARS-CoV-2 variants of concern

**Fig. 7:**
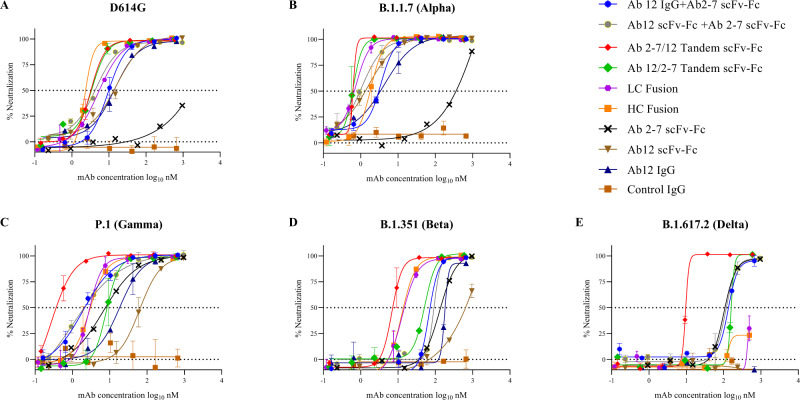
In vitro neutralization curves using GFP engineered SARS-CoV-2 variant strains. Neutralization curves were performed in triplicate using the mono- and bispecific Ab constructs against the D614G variant (**A**) and the subsequent B.1.1.7 (**B**), P.1 (**C**), B.1.351 (**D**), and B.1.617.2 (**E**) variants of concern which contain a number of additional spike mutations. The greatest effect of these mutations can be seen in the B.1.351 and B.1.617.2 neutralization plots where all curves display a substantial shift to the right. The exception to this is the Ab 2-7/Ab 12 tandem scFv-Fc, which demonstrates remarkable breadth of neutralization across the four variants of concern. Samples were tested with *n* = 3 biologically independent samples and data are presented as mean ± standard deviation. Source data are provided as a Source Data file.

In vitro neutralization experiments using the D614G, B.1.1.7, P.1, B.1.351, and B.1.617.2 variants were performed next^48^. Similar to the spike binding experiments, the D614G and B.1.1.7 variants showed comparable neutralization patterns to that of the WT strain shown previously in Figs. 1 and 5, with the BsAbs showing the greatest neutralization, Ab 12 falling in the middle, and Ab 2-7 showing the weakest neutralization (Fig. 7A, B). However, the efficacy of these two mAbs changed markedly with respect to the variants of concern. Ab 12 neutralization decreased significantly against P.1 and B.1.351, and is completely abolished against the B.1.617.2 strain. In contrast, Ab 2-7 showed a marked gain in neutralization activity against these same three strains. This is further reflected when looking at cocktail therapies against the B.1.351 and B.1.617.2 variants, as the Ab mixture generally performs at a level similar to Ab 2-7, suggesting that the inclusion of Ab 12 in the mixture is providing minimal benefit (Fig. 7C–E, Supplementary Table 3).

The BsAb advantage over cocktails is seen in the neutralization of B.1.351, as both IgG fusions and the Ab 2-7/Ab 12 tandem scFv-Fc constructs display moderate improvements in neutralization compared to a combination of parental Abs (Fig. 7D). Furthermore, the strength of the Ab 2-7/Ab 12 tandem scFv-Fc construct is further exemplified against B.1.617.2. As shown in Fig. 7E, while Ab 12 and both IgG fusions possesses no or weak neutralization ability respectively and the Ab 12/Ab 2-7 tandem mirrors that of Ab 2-7 alone, the Ab 2-7/Ab 12 tandem scFv-Fc tandem displays potent neutralization with a circa 12-fold improvement compared to the combination therapy. To evaluate if the increased neutralization of these BsAb constructs was a result of synergy or simply an additive effect of the two parental components, the neutralization curves in Fig. 6 were analyzed in CompuSyn and Combination Index (CI) scores were calculated at the IC_50_ and IC_95_^49–51^. As shown in Supplementary Fig. 10, the BsAbs and combination therapies mostly display synergistic (CI < 1) activity against the tested variants with the Ab 2-7/Ab 12 Tandem scFv-Fc construct often displaying the greatest synergy.

## Discussion

In this manuscript we characterize two neutralizing anti-SARS-CoV-2 antibodies that target non-overlapping epitopes with different binding and therapeutic efficacies both in vitro and in vivo. Class 1 Ab 12 was found to be more potent than Ab 2-7 in vitro and in vivo and triggers the spike protein to undergo an irreversible switch into the post-fusion conformation whereas Class 4 Ab 2-7 targets the conserved RBD core domain, binds with higher affinity and does not trigger spike shedding. To broaden the anti-viral properties these two mAbs, they were engineered into a series of four tetravalent, IgG-like BsAbs. The outcome of both in vitro and in vivo studies demonstrated that the format and orientation of these two binding sites were important for breadth of neutralization and in vivo protection. Compared to each mAb alone or cocktails of the mAbs in different formats, the BsAbs generally showed enhanced neutralization activity against WT and tested variants of concern (Fig. 5, 7). The Ab 2-7/Ab 12 tandem BsAb showed the most potent in vitro neutralization against WT and all variants of concern that were studied and was 12-fold more potent than the other BsAbs or the antibody cocktails against B.1.617.2.

Antibody combinations targeting multiple epitopes are becoming common practice for anti-viral therapies. The first COVID-19 combination therapies were developed by Regeneron and Lilly, with AstraZeneca’s recently completing clinical trials. The Class 1 & 2 antibody pairs developed by Lilly (etesevimab and bamlanivimab) and AstraZeneca (tixagevimab and cilgavimab) both target the ACE2 binding interface^52,53^. Conversely, like the BsAbs described in this work, Regeneron’s pair consists of an RBM directed Class 1 (casirivimab) and an RBD core targeting Class 4 (imdevimab) Ab^52^. While there is a clear neutralization benefit to targeting two regions within the ACE2 binding motif, this region is prone to the development of mutations that lead to antibody evasion like those seen at residues L452, E484, and N501^26,54,55^. The importance of epitope selection is further exemplified by the ineffectiveness of Lilly’s combination therapy against the P.1 and B.1.351 variants, and the subsequent distribution halt only months after receiving EUA, however distribution has since been resumed in selected states^56^. Ab 12 is comparable to bamlanivimab and casirivimab as it is a potent neutralizer, though sensitive to the RBD mutations seen in the B.1.351, P.1, and B.1.617.2 variants. Ab 12 was also sensitive to the Q498Y and P499T mutations in the mouse-adapted strain but these mutations are rarely seen in circulating strains^55,57^. Ab 2-7 targets the conserved region of the RBD known to confer broad neutralization and narrow viral escape profiles, and while it shows weak neutralization against D614G and B.1.1.7 there is a significant gain in potency of neutralization against B.1.351, P.1 and B.1.617.2 (Fig. 7). These results suggest that the K417/E484 mutations in the B.1.351 and P.1 strains, and L452/T478 mutations in B.1.617.2 increases their sensitivity to Ab 2-7 neutralization.

To further enhance the synergistic effect of these two mAbs, we developed four BsAbs using three different scaffolds. Due to the linked nature of the binding domains, BsAbs are known to enhance the affinity of the component mAbs by sterically hindering disassociation and artificially increasing the localized concentration of the unbound arm^58^. As seen with the original Wuhan SARS-CoV-2 strain, the BsAbs displayed moderate improvements when tested against the D614G, B.1.1.7, and P.1 variants compared to the mono-Ab therapies and less improvement over the combination cocktail therapies of the parental mAbs (Supplementary Table 3). The exception to this was the Ab 2-7/Ab 12 tandem scFv-Fc which displayed a 3.7-fold improvement compared to the scFv-Fc combination against the P.1 variant. The synergistic effect was even more pronounced with this construct against the B.1.351 and B.1.617.2 strains, with 13- and 12-fold improvement compared to the cocktail respectively. Importantly, the BsAb is only circa 2.1-fold less potent against the B.1.351 variant compared to the Regeneron cocktail which is 9.1-fold less potent^59^. The ability of the Ab 2-7/Ab 12 tandem scFv-Fc to maintain superior neutralization compared to the other BsAbs is likely due to the orientation of the binding epitopes on the RBD. The reverse orientation tandem scFv-Fc (Ab 12/Ab 2-7) is not able to enact similar synergy as the CI values are higher and the neutralization curves tend to be similar or weaker to that of the combination against the P.1 and B.1.617.2 strains. This suggests that in the Ab 12/Ab 2-7 format, the tandem scFvs are not acting in concert, rather they are binding independent of each other, thus they simply resemble their parental components. The enhanced binding and synergistic neutralization is strong against the B.1.617.2 variant, as Ab 12 alone possesses no neutralization capability, due to decreased binding similar to that seen with bamlanivimab^2^. Both IgG fusions also display minimal neutralization against this variant, suggesting that the paratopes of the Ab 2-7 scFvs displayed on these fusions are not oriented properly for synergistic binding and enhanced neutralization. In the case of the Ab 2-7/Ab 12 tandem scFv-Fc, the Ab 2-7 domain is able to provide an anchor point for Ab 12, allowing it to overcome its decreased binding efficacy to the various mutant strains, and enact potent synergistic neutralization.

To date, numerous laboratories have developed anti-SARS-CoV-2 BsAbs. Similar to the CoV-X2 BsAb developed by De Gasparo et al., our BsAbs are fully human Ig-like molecules that display potent neutralization against a wide range of SARS-CoV-2 variants^60^. Biochemical characterization of the lead Ab 2-7/12 tandem scFv-Fc and the other BsAb constructs demonstrate properties suitable for clinical development, including efficient expression and assembly, minimal aggregation, and comparable thermostability to the parental constructs (Supplementary Fig. 6A–C). Additionally, they maintain binding to the neonatal Fc receptor (FcRn), an important component to IgG recycling and extending serum half-life in vivo (Supplementary Fig. 7E). Li et al. also used a pair of non-competing antibodies to develop a dual-variable domain immunoglobulin (DVD-Ig) and IgG-scFv BsAbs, the latter of which is comparable to our HC Fusion. Their lead construct displayed similar potency against live virus, however the epitopes targeted by their Ab pair provided greater breadth of neutralization for the IgG-scFv construct^61^. Both Cho et al. and Ku et al. first identified non-competing mAb pairs that displayed limited sensitivity to the immune evading mutations to develop a series of DVD-Ig bispecifics. From this, they demonstrated that their BsAbs were able to crosslink adjacent spike proteins, a novel mechanism of neutralization that is not accessible by typical mAbs^62,63^. A major difference between CoV-X2 and the other BsAbs discussed here is that the CoV-X2 construct maintains the two binding site structure of conventional mAbs, whereas our constructs introduce two additional binding sites resulting in a tetravalent molecule allowing for increased avidity, inter-spike binding, and enhanced neutralization potential.

As the SARS-CoV-2 virus continues to evolve, innovative strategies are urgently needed to optimize Ab potency and breadth of neutralization. The commercially developed Abs described previously were derived from convalescent COVID-19 patients (Lilly/AstraZeneca) or from immunization of engineered mice (Regeneron)^24,64,65^. In contrast, our antibodies were discovered via panning of a non-immune phage library that has not undergone affinity maturation for SARS-CoV-2 spike. We previously used this pipeline during the SARS-CoV and MERS campaigns to develop therapeutic Abs and cocktails, thus demonstrating that these non-immune Ab libraries created 23 years ago contain Abs that recognize a heterogenous group of CoV spike proteins^66–69^. Interestingly, while Ab 12 activity decreased significantly against P.1 and B.1.351 strains, and was completely abolished against the B.1.617.2 strain, Ab 2-7 showed a marked gain in neutralization activity against these same three strains. Deep mutational scanning of both the RBD epitopes and the Ab binding paratopes can be performed to further interrogate the binding interaction of our antibodies, allowing for the identification of potential escape mutants and providing a means for the engineering of affinity matured Abs with enhanced neutralization capabilities^24,70,71^. Tackling this problem is not trivial for a BsAb since variable effects on synergy or antagonism may be seen; however, the Ab 2-7/Ab 12 tandem scFv-Fc exhibits synergistic properties and demonstrates potent neutralization against the currently tested variants of concern. The engineering and development of symmetric BsAbs is a rapidly growing field with significant therapeutic and manufacturing benefits. Although the tandem scFv-Fcs developed in this work provide evidence that synergistic neutralization can be achieved, further optimization can be performed to increase their breadth of binding and potency. Accordingly, they are an important steppingstone and serve as a foundation for the future development of a unique scaffold for broadly reactive BsAbs against emerging variants of concern.

## Methods

### Phage panning

Peripheral B cells from 57 healthy donors were used to create two, non-immunized scFv-phage libraries totaling 2.7 × 10^10^ members. 1.66 × 10^12^ pfu of scFv-phage from each library was combined and used to perform 3 rounds of panning against SARS-CoV-2 S1 protein (Sino Biologicals) or SARS-CoV-2 RBD protein expressed in our lab. Briefly, SARS-CoV-2 RBD or S1 proteins were passively absorbed onto Nunc MaxiSorp Immuno tubes (Thermo Fisher Scientific) overnight in PBS. Coated tubes were incubated with the phage library, followed by extensive PBS/PBS-T (PBS + 0.05% Tween-20) washes to remove nonspecific phage. Bound phage were eluted with 100 mM triethylamine and neutralized with 1 M Tris-HCl, pH 7.5. The eluted phage solution was neutralized, amplified, and used for further selection or screening. SARS-CoV-2 S1 and RBD coating concentration was decreased in each round to increase the affinity of the enriched antibodies.

Screening of the enriched library was performed by selecting circa 1300 bacterial colonies from the 3rd round of panning and culturing them in individual wells in 96 well plates. Small-scale rescue was performed via VCS-M13 helper phage and the phage supernatant was used to screen via SARS-CoV-2 RBD or S1 coated ELISA plates. Positive wells were selected for colony PCR and subsequent sanger sequencing. Unique sequences were then cloned into the appropriate expression vector for further analysis.

### BsAb design

BsAbs were designed to utilize different functional formats of Abs 12 and 2-7. IgG fusions were built using Ab 12 IgG as the scaffold, with Ab 2-7 scFv genetically fused to the C terminus of the CL (LC fusion) or CH3 (HC fusion) domains via a flexible (G4S)5 or (G4S)2 linker respectively. Tandem scFv-Fc construct consists of two scFvs linked with a flexible (G4S)3 linker fused to a CD8-hinge – IgG1-Fc domains and was created in both orientations (Ab 12/Ab 2-7 and Ab 2-7/Ab 12).

### Recombinant SARS-CoV-2 protein production

hACE2 (transOMIC) and SARS-CoV-2 RBD/S1 (Sino Biologics) cDNA were purchased and cloned into our mammalian expression vector. Stabilized SARS-CoV-2 spike trimer expressing plasmid was obtained through BEI and the HexaPro expression plasmid was a kind gift from Dr. Jason McLellan’s Lab (UT Austin). All proteins were expressed in the Expi293F system and cells were transiently transfected by Expifectamine 293 (all components purchased from Thermo Fisher Scientific) following the standard protocol. 4-5 days after transfection, supernatants were clarified and incubated with Ni-NTA resin (Qiagen) overnight at 4 °C. They were subsequently purified via gravity flow column and buffer exchanged by centrifugation in Amicon centrifugal filters. Avi tagged proteins were biotinylated by Avidity’s BirA biotiniylation kit following standard protocols. Protein concentration was measured on a Nanodrop 100 using the MW and extinction coefficients calculated on ExPASy’s ProtParam.

### Antibody production

Antibodies were produced in Expi293F cells (Thermo Fisher Scientific catalog #A14635) using transiently transfected mammalian expression vectors following the standard Expifectamine293 protocol. The harvested supernatants were incubated with Protein A-Sepharose 4B resin (Invitrogen) overnight at 4 °C followed by purification via gravity flow columns (BioRad) and buffer exchanged by centrifugation in Amicon centrifugal filters. Protein concentration was measured on a Nanodrop 100 using the MW and extinction coefficients calculated on ExPASy’s ProtParam.

### Biolayer interferometry (BLI) binding assays

All assays were performed in 96-well black plates on an Octet Red96 instrument (Sartorius) with shaking at 1000 RPM. Sensors (SA for kinetics, competition, and FcRn; AHC for variant spike binding) were first loaded with the ligand of interest, then transferred to wells containing the appropriate analyte diluted in PBST to determine *k*_on_ followed by wells containing only PBST to measure k_off_. For sensors that were used with multiple analytes, regeneration was performed by alternating cycles of 0.1 M glycine buffer pH 2.7 and PBST. All samples were made in PBST except for FcRn binding assay, which was prepared in PBS titrated to pH 6 with hydrochloric acid. Naked sensors or sensors loaded with an irrelevant protein were used as nonspecific binding controls. To enable accurate curve fitting, data preprocessing was performed using built-in functions of the Octet Data Analysis Software v10.0.0.5. First, nonspecific binding (reference wells) was subtracted from the dataset, followed by *Y*-axis alignment to the baseline, inter-step correction to remove step misalignments due to assay artifacts, and Savitzky-Golay filtering to remove high-frequency noise. The Octet Data Analysis Software (v10.0.0.5) was then used for kinetic curve fitting of the processed data using a 1:1 binding and a global fit of the tested concentrations.

### FACS S1 disassociation

FACS S1 disassociation assay was previously described in Wec et al.^23^. Briefly, 293T-Spike cells (293T-17 ATCC catalog #CRL-11268 transduced to stably express the SARS-CoV-2 spike) were washed, resuspended at 4E6 cells/ml in MACS buffer with 20 uM cycloheximide (MACS+) to inhibit protein synthesis, and aliquoted at 50 ul per well in a V bottom 96 well plate^72^. Abs were diluted to 200 nM in MACS+ and both Ab dilution and cell plates were incubated separately at 37 °C for 15 min to equilibrate the plates. At the desired time points, 50 ul of Ab dilution was transferred to the corresponding well in the 96 well plate and mixed via pipetting. The plate was maintained at 37 °C during the entire time course. After the last timepoint, the cell plates were rapidly transferred to ice and quenched with ice cold MACS buffer. The plate was washed 2x with MACS buffer, followed by resuspension in anti-hFc-APC (BioLegend clone HP6017) at the manufacturer’s recommended concentration for 20 min at 4 °C. Cells were washed 3x with MACS buffer before fixation by 1% paraformaldehyde (Boston Bio Products). Cells were analyzed on a BD Canto II with HTS reader. Samples were run in duplicate.

### Production of Ab 2-7 scFv for cryo-electron microscopy

A plasmid encoding for the Ab 2-7 single-chain Fv was transiently transfected into HEK293F cells (Thermo Fisher Scientific catalog #R79007). Six days after transfection, the medium was harvested, buffer exchanged, and concentrated, then passed over Nickel NTA affinity resin. Elution fractions containing Ab 2-7 scFv were pooled, concentrated, and further purified by size exclusion chromatography (Superdex 200, 2 mM Tris pH 8, 200 mM NaCl, 0.02% [w/v] sodium azide). Elution fractions containing monomeric, monodisperse Ab 2-7 scFv were pooled, concentrated, aliquoted, and flash frozen in liquid nitrogen.

### Cryo-EM of Ab 2-7 complexed with SARS-CoV-2 S D614G

Purified Ab 2-7 scFv was mixed with purified SARS-CoV-2 S D614G (ExcellGene SA) at a molar ratio of 1.5:1 (Ab 2-7 to spike). Spike protein was purified by size exclusion prior to complex formation to remove soluble aggregates. The sample was incubated at room temperature for 30 min, deposited onto gold cryo-EM grids (UltrAuFoil 1.2/1.3), which had been plasma cleaned for 3 min using a Solarus 950 plasma cleaner (Gatan) with a 4:1 ratio of O2/H2. The excess protein sample was blotted away (Vitrobot filter paper; -3 force, 4 s blot time, 100% humidity, room temperature) before plunge freezing in liquid ethane (Vitrobot, ThermoFisher). Grids were screened for quality on a Talos F200C transmission electron microscope equipped with a Ceta 16 M detector (ThermoFisher). Grids that passed quality control were loaded onto a Titan Krios (ThermoFisher) operating at 300 kV, equipped with a K3 Detector (Gatan). The pixel size was 0.66 Å. Motion correction and CTF estimation were performed in WARP. Particle picking, 2D classification, 3D reconstruction and refinement were performed in cryoSPARC. Model building was performed iteratively using Coot, Isolde, and Phenix. A detailed description of image collection, processing and model quality is shown in Supplementary Figs. 2, 3.

### Cryo-electron microscopy of the complex between SARS-CoV-2 S-D614G and Ab 12 Fab

0.5 mg of Prefusion-stabilized SARS-CoV-2-D614G was incubated with a 10-fold molar excess of Ab 12 Fab overnight. The complex was purified by size exclusion chromatography on a Superose 6i 10/300 GL column and concentrated to 2 mg/ml. Electron microscopy grids were prepared by placing a 3 ul aliquot of the sample on 2/1 C-flat grids (2/1C-3T, Protochips Inc) glow discharged for 15 s on a Pelco glow discharge unit, and frozen by dipping in liquid ethane using a Vitrobot Mark IV device, after a 6 s blotting time (Whatman #1 filter paper). The grids were then clipped and transferred to the autoloader system of a Titan Krios G3 electron microscope (ThermoFisher Scientific) equipped with a K3 direct electron detector (Gatan Inc) at the end of a BioQuantum energy filter, using an energy slit of 20 eV. The microscope was operated with an accelerating voltage of 300 kV, a total electron dose of 50e/A^2^. Grids were imaged at a magnification of 75kX, corresponding to a pixel size of 0.66 with the program EPU, under zero loss imaging, using a 20 eV window. Motion correction, CTF estimation, and particle picking were done with Warp. Extracted particles were exported to cryoSPARC-v2 (Structura Biotechnology Inc.) for 2D classification, ab initio 3D reconstruction, and refinement. C1 symmetry was used during homogeneous refinement. Models were docked into the experimental EM density using Chimera and Phenix. One starting model was used: SARS-CoV-2 S with two RBDs in the “up” conformation (PDB ID 7K8T, 10.1038/s41586-020-2852-1), and a homology model of Ab12 Fab that was generated using the SAbPred server.

### Image processing for Ab 12

We collected 3979 images which were pre-processed using the program Warp, which does motion correction, followed by CTF estimation and particle picking using a machine learning algorithm^73^. In this way, we ended up with 676,973 particles, which were transferred to the program cryosparc for further processing^74^. The processing started with the use of a blob picker, followed by 2D classification to discard bad particles, followed by an ab initio reconstruction to produce a suitable volume for further refinement. For the ab initio we requested three different classes, using a maximum of 20,000 particles each, and imposing 3-fold symmetry.

Refinement for each of the ab initio classes was done without imposing any symmetry and using all the particles remaining in the dataset. The reconstruction that was deemed to be the best one by appearance was further used to produce three different classes by heterogeneous refinement, using the volumes produced by the homogeneous refinements. The class that gave the best reconstruction by appearance was then used for non-uniform reconstruction. This reconstruction was re-projected and the projections used to search for more particles using the template picker. In this way, we ended up with a total of 293,289 particles. With these new particles, we ran a homogeneous refinement using the last reconstruction for alignment, followed by a cycle of heterogeneous refinement, and then a non-uniform refinement. We were aiming to improve the visualization of the details in the Fab regions. The final reconstruction has a total of 131,548 particles.

### Plaque reduction neutralization test

A series of 10 half-log dilutions was prepared in triplicate for each antibody or antibody mixture in Dulbecco’s Phosphate Buffered Saline (DPBS) (Gibco). Each dilution was incubated at 37 °C and 5% CO_2_ for 1 hour with an equal volume of 1000 plaque forming units/ml (PFU/ml) of SARS-CoV-2 (isolate USA‐WA1/2020), diluted in Dulbecco’s Modified Eagle Medium (DMEM) (Gibco) containing 2% fetal bovine serum (Gibco) and antibiotic-antimycotic (Gibco). Controls included DMEM containing 2% fetal bovine serum and antibiotic-antimycotic only as a negative control, 1000 PFU/ml SARS-CoV-2 incubated with DPBS, and 1000 PFU/ml SARS-CoV-2 incubated with DMEM. Two hundred microliters of each dilution or control were added to confluent monolayers of Vero E6 cells (BEI Resources catalog #NR-596) in triplicate and incubated for 1 hour at 37 °C and 5% CO_2_. The plates were gently rocked every 5–10 min to prevent monolayer drying. The monolayers were then overlaid with a 1:1 mixture of 2.5% Avicel® RC‐591 microcrystalline cellulose and carboxymethylcellulose sodium (DuPont Nutrition & Biosciences, Wilmington, DE) and 2X Modified Eagle Medium (Temin’s modification, Gibco) supplemented with 2X antibiotic‐antimycotic (Gibco), 2X GlutaMAX (Gibco) and 10% fetal bovine serum (Gibco). Plates were incubated at 37 °C and 5% CO_2_ for 2 days. The monolayers were fixed with 10% neutral buffered formalin and stained with 0.2% aqueous Gentian Violet (RICCA Chemicals, Arlington, TX) in 10% neutral buffered formalin for 30 min, followed by rinsing and plaque counting. The half maximal inhibitory concentrations (IC50) were calculated using GraphPad Prism 8.

### nLuc virus

Vero E6 cells (USAMRIID) were plated at 20,000 cells per well in black-walled 96-well plates (Corning 3904). mAbs were serially diluted 3-fold with a maximum of eight dilution spots. Diluted antibodies were mixed with 85 PFU/well of recombinant SARS-CoV-2-nLuc virus, and the mixtures were incubated at 37 °C with 5% CO_2_ for 1 hour. Following incubation, growth media was removed, and virus-antibody mixtures were added to the cells in duplicate. Virus-only controls were included in each plate. Following infection, plates were incubated at 37 °C with 5% CO_2_ for 48 hours. After the 48-hour incubation, cells were lysed, and luciferase activity was measured via Nano-Glo Luciferase Assay System (Promega) according to the manufacturer specifications. Neutralization titers were defined as the sample dilution at which a 50% reduction in relatively light unit (RLU) was observed relative to the average of the virus control wells.

### Serum In Vitro Neutralization Assay

Aliquots of mNeonGreen reporter SARS-CoV-2 were pre-incubated for 1 hour in 5% CO_2_ at 37 °C with serial 2-fold dilutions of serum and inoculated into Vero-E6 (ATCC catalog #CRL-1586) triplicate monolayers in black polystyrene 96-well plates with clear bottoms (Corning). The final amount of virus was 200 PFU/well, serum was diluted with an initial 1:20 dilution followed by 2x fold dilutions. Cells were maintained in Minimal Essential Medium (ThermoFisher Scientific) supplemented by 10% FBS (HyClone) and 0.1% genamicin in 5% CO_2_ at 37 °C. After 2 days of incubation, fluorescence intensity of infected cells was measured at a 488 nm wavelength using a Cytation 5 Cell Imaging Multi-Mode Reader (Biotek). The signal readout was normalized to virus control aliquots with no serum added and was presented as the percentage of neutralization.

### In vitro live virus neutralization assays

50 ul of mNeonGreen reporter SARS-CoV-2 viruses were pre-incubated for 1 hour in 5% CO_2_ at 37 °C with 50 ul of serial 4-fold dilutions of antibodies and inoculated into Vero-E6 (ATCC catalog #CRL-1586) triplicate monolayers in black polystyrene 96-well plates with clear bottoms (Corning). The final amount of virus was 200 PFU/well, initial concentration of antibodies was 200 ug/ml. Cells were maintained in Minimal Essential Medium (ThermoFisher Scientific) supplemented by 10% FBS (HyClone) and 0.1% genamicin in 5% CO_2_ at 37 °C. After 2 days of incubation, fluorescence intensity of infected cells was measured at a 488 nm wavelength using a Cytation 7 Cell Imaging Multi-Mode Reader (Biotek). The signal readout was normalized to virus control aliquots with no antibodies added and was presented as the percentage of neutralization.

### Viruses

The SARS-CoV-2 challenge strain used in this study is the first U.S. isolate SARS-CoV-2 USA-WA1/2020 from the Washington State patient identified on January 22, 2020^75^. Passage 3 was obtained from the World Reference Center for Emerging Viruses and Arboviruses (WRCEVA) at UTMB. Virus stocks were propagated in Vero E6 cells. The challenge stock used in this study is passage 5. The recombinant SARS-CoV-2 expressing Neon Green protein (SARS-CoV-2-mNG) and its Spike protein mutants used in the neutralization assay was developed by Dr. Pei-Yong Shi at UTMB^48,76^. Virus stocks were propagated in Vero E6 cells and a passage 1 was used in this study.

The Alpha variant virus incorporates the following substitutions: Del 69-70, Del 144, E484K, N501Y, A570D, D614G, P681H, T716I, S982A, D1118H. The Beta variant incorporates the following substitutions: Del 24, Del 242-243, D80A, D215G, K417N, E484K, N501Y, D614G, H665Y, T1027I. The Delta variant incorporates the following substitutions: T19R, G142D, Del 156-157, R158G, L452R, T478K, D614G, P681R, Del 689-691, D950N; the deletion at positions 689-691 has not been observed in nature and was identified upon one passage of the virus. The Gamma variant incorporates the following substitutions: L18F, T20N, P26S, D138Y, R190S, K417T, E484K, N501Y, D614G, H655Y, T1027I.

### Syrian golden hamster experiments

Animal challenge studies were conducted in the ABSL-4 facility of the Galveston National Laboratory. The animal protocol for testing of mAbs in mice was approved by the Institutional Animal Care and Use Committee (IACUC) of the University of Texas Medical Branch at Galveston (UTMB).

Female Syrian golden hamsters (6-7 weeks old) were microchipped the day before challenge for identification and temperature monitoring. On day 0, hamsters were anesthetized with ketamine/xylazine and challenged with SARS-CoV-2 by the intranasal (IN) route 1 × 10^5^ PFU diluted in sterile PBS. Body weight and body temperature were measured each day, starting at day 0.

On day 1 post-challenge (dpc) hamsters were treated with 5 mg/kg of monoclonal antibodies diluted in 0.5 ml of sterile PBS via intraperitoneal route (IP). The control group received an equal volume of sterile PBS via the same route. At 3 dpc, all animals were euthanized. At necropsy, terminal serum was collected from all animals. Lungs were harvested for all groups.

### Syrian golden hamster tissue processing and viral load determination

For the pathogenicity study, animals from each study group were euthanized at 3 dpc, and the lungs were harvested. Right lungs were placed in L15 medium supplemented with 10% fetal bovine serum (Gibco) and Antibiotic-Antimycotic solution (Gibco), flash-frozen in dry ice and stored at -80 °C until processing. Tissues were thawed and homogenized using the TissueLyser II system (Qiagen). Tissue homogenates were titrated on Vero E6 cell monolayers in 96-well plates to determine viral loads. 10-fold dilutions of the lung supernatants were incubated for 1 hour and replaced with 100 µL of 0.9% methylcellulose in minimal essential medium (MEM) containing 10% fetal bovine serum (Quality Biologicals) and 0.1% gentamicin sulfate (Mediatech), followed by incubation at 37 °C. Plates were fixed with 10% buffered formalin (Thermo Fisher) and removed from BSL-4. Foci were visualized by staining monolayers with a mixture of 37 SARS-CoV-2 specific human antibodies kindly provided by Distributed Bio. HRP-labeled goat anti-human IgG (SeraCare) was used as the secondary antibody at a dilution of 1:500. Primary and secondary antibodies were diluted in 1X DPBS with 5% milk. Assays were developed with AEC substrate (enQuire Bioreagents).

### Syrian golden hamster histopathology

During necropsy, gross lesions were noted and representative lung tissues from the left lobe were collected in 10% formalin. After a 24-hour initial fixation at 4 °C, the lung tissues were transferred to fresh 10% formalin for an additional 48-hour fixation, prior to removal from containment. Formalin-fixed tissues were processed by standard histological procedures by the UTMB Anatomic Pathology Core. About 4 μm-thick sections were cut and stained with hematoxylin and eosin (HE). Sections of lungs were examined for the extent of inflammation, type of inflammatory foci, and changes in alveoli/alveolar septa/airways/blood vessels in parallel with sections from uninfected or control animals. The blinded tissue sections were semi-quantitatively scored for pathological lesions using the criteria described in Supplementary Table 1. Significance was assessed using a Kruskall–Wallis test with Dunn’s post-hoc correction.

### Prophylactic efficacy in mouse models

Eleven to 12-month-old female BALB/c mice (BALB/c AnNHsd, Envigo, stock# 047) were used for mouse-adapted SARS-CoV-2 (SARS-CoV-2 MA) in vivo protection experiments as described previously^37^. Ten-week-old HFH4-hACE2 transgenic mice were used for SARS-CoV-2 WT in vivo protection experiments^37,77^. For evaluating the prophylactic efficacy of single mAbs and mAb combinations, mice were injected intraperitoneally (ip) with the appropriate concentration of each mAb combination 12 hours prior to infection. Mice were infected intranasally with 1 × 10^5^ PFU SARS-CoV-2 MA or SARS-CoV-2 WT, respectively. At 48 hours post-infection, mice were euthanized, and lung tissue was harvested for viral titer as measured by plaque assays. For viral plaque assays, the caudal lobe of the right lung was homogenized in PBS, and the tissue homogenate was then serial-diluted onto confluent monolayers of Vero E6 cells, followed by agarose overlay. Plaques were visualized with overlay of Neutral Red dye on day 2 post-infection. All mouse studies were performed at the University of North Carolina (Animal Welfare Assurance #A3410-01) using protocols (19-168) approved by the UNC Institutional Animal Care and Use Committee (IACUC) and were performed in a BSL3 facility at UNC.

### Reporting summary

Further information on research design is available in the Nature Research Reporting Summary linked to this article.

## Supplementary information

Supplementary Information

Reporting Summary

## Data Availability

The atomic models generated in this study have been deposited into the PDB with accession number 7T3M (spike with 3 Ab 2-7 scFvs) and 7T67 (apo spike trimer). The corresponding cryo-EM density maps generated in this study have been deposited into the Electron Microscopy Data Bank with accession numbers EMD-25689 and EMD-25663 (Spike complexed with Ab 2-7 scFv and), EMD-25690 (RBD:scFv subcomplex), EMD-25711 (unbound spike), EMD-25618 (Spike complexed with Ab 12). Additional data generated in this study are provided in the Source Data file or available from the corresponding author upon reasonable request. Source data are provided with this paper.

## Source data

Source Data

## References

[1] Lambrou, A. S. et al. Implementation of SARS-CoV-2 monoclonal antibody infusion sites at three medical centers in the United States: strengths and challenges assessment to inform COVID-19 pandemic and future public health emergency use. *medRxiv* **6**, 10.1101/2021.04.05.21254707 (2021). 10.1017/dmp.2022.15 PMC9002153 35027098

[2] Planas D (2021). Reduced sensitivity of SARS-CoV-2 variant Delta to antibody neutralization. Nature.

[3] Merchant AM (1998). An efficient route to human bispecific IgG. Nat. Biotechnol..

[4] Orcutt KD (2010). A modular IgG-scFv bispecific antibody topology. Protein Eng. Des. Sel..

[5] Boado RJ, Lu JZ, Hui EKW, Pardridge WM (2010). IgG-single chain Fv fusion protein therapeutic for Alzheimer’s disease: expression in CHO cells and pharmacokinetics and brain delivery in the Rhesus monkey. Biotechnol. Bioeng..

[6] Steinmetz A (2016). CODV-Ig, a universal bispecific tetravalent and multifunctional immunoglobulin format for medical applications. MAbs.

[7] Holliger P, Prospero T, Winter G (1993). ‘Diabodies’: small bivalent and bispecific antibody fragments. Proc. Natl Acad. Sci. USA.

[8] Veri MC (2010). Therapeutic control of B cell activation via recruitment of Fcγ receptor IIb (CD32B) inhibitory function with a novel bispecific antibody scaffold. Arthritis Rheum..

[9] Ahmad, Z. A. et al. ScFv antibody: Principles and clinical application. *Clin. Dev. Immunol*. **2012**, 980250 (2012). 10.1155/2012/980250 PMC3312285 22474489

[10] Ho, D. 10E8.4/iMab Bispecific Antibody in HIV-uninfected and HIV-infected Adults (NCT03875209) (2019).

[11] Bohac, C. MGD014 in HIV-Infected Individuals on Suppressive Antiretroviral Therapy (NCT03570918) (2018).

[12] Lu RM (2020). Development of therapeutic antibodies for the treatment of diseases. J. Biomed. Sci..

[13] Bournazos S, Gazumyan A, Seaman MS, Nussenzweig MC, Ravetch JV (2016). Bispecific Anti-HIV-1 antibodies with enhanced breadth and potency. Cell.

[14] Moshoette T, Ali SA, Papathanasopoulos MA, Killick MA (2019). Engineering and characterising a novel, highly potent bispecific antibody iMab-CAP256 that targets HIV-1. Retrovirology.

[15] Zanin M (2015). An anti-H5N1 influenza virus FcDART antibody is a highly efficacious therapeutic agent and prophylactic against H5N1 influenza virus infection. J. Virol..

[16] Frei JC (2016). Bispecific antibody affords complete post-exposure protection of mice from both Ebola (Zaire) and Sudan viruses. Sci. Rep..

[17] Yuan M (2020). A highly conserved cryptic epitope in the receptor-binding domains of SARS-CoV-2 and SARS-CoV. Science.

[18] Marra MA (2003). The Genome sequence of the SARS-associated coronavirus. Science.

[19] Pang NYL, Pang ASR, Chow VT, Wang DY (2021). Understanding neutralising antibodies against SARS-CoV-2 and their implications in clinical practice. Mil. Med. Res..

[20] Jin D, Wei J, Sun J (2021). Analysis of the molecular mechanism of SARS-CoV-2 antibodies. Biochem. Biophys. Res. Commun..

[21] Niu L, Wittrock KN, Clabaugh GC, Srivastava V, Cho MW (2021). A structural landscape of neutralizing antibodies against SARS-CoV-2 receptor binding domain. Front. Immunol..

[22] Gavor E, Choong YK, Er SY, Sivaraman H, Sivaraman J (2020). Structural basis of SARS-CoV-2 and SARS-CoV antibody interactions. Trends Immunol..

[23] Wec AZ (2020). Broad neutralization of SARS-related viruses by human monoclonal antibodies. Science.

[24] Greaney AJ (2021). Complete mapping of mutations to the SARS-CoV-2 spike receptor-binding domain that escape antibody recognition. Cell Host Microbe.

[25] Dong J (2021). Genetic and structural basis for SARS-CoV-2 variant neutralization by a two-antibody cocktail. Nat. Microbiol..

[26] Harvey WT (2021). SARS-CoV-2 variants, spike mutations and immune escape. Nat. Rev. Microbiol..

[27] Hastie KM (2021). Defining variant-resistant epitopes targeted by SARS-CoV-2 antibodies: a global consortium study. Science.

[28] Tortorici MA (2021). Broad sarbecovirus neutralization by a human monoclonal antibody. Nature.

[29] Sia SF (2020). Pathogenesis and transmission of SARS-CoV-2 in golden hamsters. Nature.

[30] Imai M (2020). Syrian hamsters as a small animal model for SARS-CoV-2 infection and countermeasure development. Proc. Natl Acad. Sci..

[31] Denison MR, Graham RL, Donaldson EF, Eckerle LD, Baric RS (2011). Coronaviruses: an RNA proofreading machine regulates replication fidelity and diversity. RNA Biol..

[32] Gribble J (2021). The coronavirus proofreading exoribonuclease mediates extensive viral recombination. PLoS Pathog..

[33] Pachetti M (2020). Emerging SARS-CoV-2 mutation hot spots include a novel RNA-dependent-RNA polymerase variant. J. Transl. Med..

[34] Robson F (2020). Coronavirus RNA proofreading: molecular basis and therapeutic targeting. Mol. Cell.

[35] Miao, M., De Clercq, E. & Li, G. Genetic diversity of sars-cov-2 over a one-year period of the covid-19 pandemic: a global perspective. *Biomedicines* **9**, 412 (2021). 10.3390/biomedicines9040412 PMC8069977 33920487

[36] Lythgoe, K. A. et al. SARS-CoV-2 within-host diversity and transmission. *Science* **372**, eabg0821 (2021). 10.1126/science.abg0821 PMC8128293 33688063

[37] Dinnon KH (2020). A mouse-adapted model of SARS-CoV-2 to test COVID-19 countermeasures. Nature.

[38] Gobeil SMC (2021). D614G mutation alters SARS-CoV-2 spike conformation and enhances protease cleavage at the S1/S2 junction. Cell Rep..

[39] Ozono, S. et al. SARS-CoV-2 D614G spike mutation increases entry efficiency with enhanced ACE2-binding affinity. *Nat. Commun*. **12**, 848 (2021). 10.1038/s41467-021-21118-2 PMC7870668 33558493

[40] Zhang J (2021). Structural impact on SARS-CoV-2 spike protein by D614G substitution. Science.

[41] Tracking SARS-CoV-2 variants. *World Health Organization* https://www.who.int/en/activities/tracking-SARS-CoV-2-variants/ (2021).

[42] Lazarevic, I., Pravica, V., Miljanovic, D. & Cupic, M. Immune evasion of sars‐cov‐2 emerging variants: What have we learnt so far? *Viruses* **13**, 1192 (2021). 10.3390/v13071192 PMC8310325 34206453

[43] Tzou PL, Tao K, Kosakovsky Pond SL, Shafer RW (2022). Coronavirus Resistance Database (CoV-RDB): SARS-CoV-2 susceptibility to monoclonal antibodies, convalescent plasma, and plasma from vaccinated persons. PLoS ONE.

[44] Graham C (2021). Neutralization potency of monoclonal antibodies recognizing dominant and subdominant epitopes on SARS-CoV-2 Spike is impacted by the B.1.1.7 variant. Immunity.

[45] Planas D (2021). Sensitivity of infectious SARS-CoV-2 B.1.1.7 and B.1.351 variants to neutralizing antibodies. Nat. Med..

[46] Wang P (2021). Antibody resistance of SARS-CoV-2 variants B.1.351 and B.1.1.7. Nature.

[47] Hoffmann M (2021). SARS-CoV-2 variants B.1.351 and P.1 escape from neutralizing antibodies. Cell.

[48] Liu, Y. et al. Distinct neutralizing kinetics and magnitudes elicited by different SARS-CoV-2 variant spikes. *bioRxiv* 1–22 10.1101/2021.09.02.458740 (2021).

[49] Chou, T.-C. & Martin, N. CompuSyn software for drug combinations and for general dose- effect analysis, and user’s guide. ComboSyn, Inc. Paramus, NJ 2007. www.combosyn.com (2007).

[50] Baba TW (2000). Human neutralizing monoclonal antibodies of the IgG1 subtype protect against mucosal simian–human immunodeficiency virus infection. Nat. Med..

[51] Chou T-C (2018). The combination index (CI < 1) as the definition of synergism and of synergy claims. Synergy.

[52] Kumar S, Chandele A, Sharma A (2021). Current status of therapeutic monoclonal antibodies against SARS-CoV-2. PLOS Pathog..

[53] Corti D, Purcell LA, Snell G, Veesler D (2021). Tackling COVID-19 with neutralizing monoclonal antibodies. Cell.

[54] Liu Z (2021). Identification of SARS-CoV-2 spike mutations that attenuate monoclonal and serum antibody neutralization. Cell Host Microbe.

[55] Cyrus Maher, M. et al. Predicting the mutational drivers of future SARS-CoV-2 variants of concern. *medRxiv*10.1101/2021.06.21.21259286 (2021). 10.1126/scitranslmed.abk3445 PMC8939770 35014856

[56] Hinton, D. *Emergency Use Authorization 094*. *FDA* vol. 26 (2021).

[57] Massacci A (2020). Design of a companion bioinformatic tool to detect the emergence and geographical distribution of SARS-CoV-2 Spike protein genetic variants. J. Transl. Med..

[58] Zhou HX (2003). Quantitative account of the enhanced affinity of two linked scFvs specific for different epitopes on the same antigen. J. Mol. Biol..

[59] Tada, T. et al. Decreased neutralization of SARS-CoV-2 global variants by therapeutic anti-spike protein monoclonal antibodies. *bioRxiv*10.1101/2021.02.18.4318977 (2021).

[60] De Gasparo R (2021). Bispecific IgG neutralizes SARS-CoV-2 variants and prevents escape in mice. Nature.

[61] Li Z (2022). An engineered bispecific human monoclonal antibody against SARS-CoV-2. Nat. Immunol..

[62] Cho, H. et al. Bispecific antibodies targeting distinct regions of the spike protein potently neutralize SARS-CoV-2 variants of concern. *Sci. Transl. Med*. **13**, eabj5413 (2021). 10.1126/scitranslmed.abj5413 PMC8651051 34519517

[63] Ku, Z. et al. Engineering SARS-CoV-2 cocktail antibodies into a bispecific format improves neutralizing potency and breadth. *bioRxiv*10.1101/2022.02.01.478504 (2022). 10.1038/s41467-022-33284-y PMC9499943 36138032

[64] Jones, B. E. *e*t al. LY-CoV555, a rapidly isolated potent neutralizing antibody, provides protection in a non-human primate model of SARS-CoV-2 infection. *bioRxiv* 1–29 10.1101/2020.09.30.318972 (2020).

[65] Hansen J (2020). Studies in humanized mice and convalescent humans yield a SARS-CoV-2 antibody cocktail. Science.

[66] Sui J (2004). Potent neutralization of severe acute respiratory syndrome (SARS) coronavirus by a human mAb to S1 protein that blocks receptor association. Proc. Natl Acad. Sci..

[67] Sui J (2005). Evaluation of human monoclonal antibody 80R for immunoprophylaxis of severe acute respiratory syndrome by an animal study, epitope mapping, and analysis of spike variants. J. Virol..

[68] Sui J (2009). Structural and functional bases for broad-spectrum neutralization of avian and human influenza A viruses. Nat. Struct. Mol. Biol..

[69] Tang, X. C. et al. Identification of human neutralizing antibodies against MERS-CoV and their role in virus adaptive evolution. *Proc. Natl. Acad. Sci*. **111**, E2018-26 (2014). 10.1073/pnas.1402074111 PMC4024880 24778221

[70] Starr TN (2021). Prospective mapping of viral mutations that escape antibodies used to treat COVID-19. Science.

[71] Starr TN (2020). Deep mutational scanning of SARS-CoV-2 receptor binding domain reveals constraints on folding and ACE2 binding. Cell.

[72] Beverly, L. J., Lockwood, W. W., Shah, P. P., Erdjument-Bromage, H. & Varmus, H. Ubiquitination, localization, and stability of an anti-apoptotic BCL2-like protein, BCL2L10/BCLb, are regulated by Ubiquilin1. *Proc. Natl. Acad. Sci. USA* **109**, E119–E126 (2012). 10.1073/pnas.1119167109 PMC3271887 22233804

[73] Tegunov D, Cramer P (2019). Real-time cryo-electron microscopy data preprocessing with Warp. Nat. Methods.

[74] Punjani A, Rubinstein JL, Fleet DJ, Brubaker MA (2017). CryoSPARC: Algorithms for rapid unsupervised cryo-EM structure determination. Nat. Methods.

[75] Harcourt J (2020). Severe acute respiratory syndrome coronavirus 2 from patient with coronavirus disease, United States. Emerg. Infect. Dis..

[76] Xie X (2020). An Infectious cDNA Clone of SARS-CoV-2. Cell Host Microbe.

[77] Hou YJ (2020). SARS-CoV-2 reverse genetics reveals a variable infection gradient in the respiratory tract. Cell.

