# Supplementary material for: IgG-like bispecific antibodies with potent and synergistic neutralization against circulating SARS-CoV-2 variants of concern
Source: Nat Commun. 2022 Oct 3;13:5814. doi: 10.1038/s41467-022-33030-4 (PMC9528872; doi:10.1038/s41467-022-33030-4)
Supplement: Supplementary file 1 — Supplementary Information [file 41467_2022_33030_MOESM1_ESM.pdf]

Title: IgG-like bispecific antibodies with potent and synergistic neutralization against circulating SARS-CoV-2 variants of concern

Author List: Matthew R. Chang[1], Luke Tomasovic[1], Natalia A. Kuzmina[2,3,4], Adam J. Ronk[2,3,4], Patrick O. Byrne[5], Rebecca Johnson[6], Nadia Storm[6], Eduardo Olmedillas[7], Yixuan J. Hou[8], Alexandra Schäefer[8], Sarah R. Leist[8], Longping V. Tse[8], Hanzhong Ke[1,9], Christian Coherd[1], Katrina Nguyen[1], Maliwan Kamkaew[1], Anna Honko[6], Quan Zhu[1,9], Galit Alter[10], Erica Ollmann Saphire[7], Jason McLellan[5], Anthony Griffiths[6], Ralph S. Baric[8], Alexander Bukreyev[2,3,4], and Wayne A. Marasco[1,9*]

Affiliations:
[1]Department of Cancer Immunology & Virology, Dana-Farber Cancer Institute, Boston, MA, 02115, USA.
[2]Department of Pathology, University of Texas Medical Branch, Galveston, TX 77555, USA.
[3]Galveston National Laboratory, Galveston, TX 77555, USA.
[4]Department of Microbiology and Immunology, University of Texas Medical Branch, Galveston, TX 77555, USA.
[5]Department of Molecular Biosciences, University of Texas, Austin, TX 78712, USA.
[6]Department of Microbiology and National Emerging Infectious Diseases Laboratories, Boston University, School of Medicine, Boston, MA, 02118, USA.
[7]La Jolla Institute for Immunology, La Jolla, CA 92037, USA.
[8]Department of Epidemiology, University of North Carolina at Chapel Hill, Chapel Hill, NC, 27599, USA.
[9]Department of Medicine, Harvard Medical School, Boston, MA, 02115, USA.
[10]Ragon Institute of MGH, MIT and Harvard, Cambridge, MA 02139, USA.

Corresponding Author:
Wayne A. Marasco
450 Brookline Ave, Smith 534A
Boston, MA 02115
Phone: 617-632-2153
Email: Wayne_Marasco@dfci.harvard.edu

Supplementary Material

Supplementary Figures 1-10

Supplementary Tables 1-3

Supplementary References

A

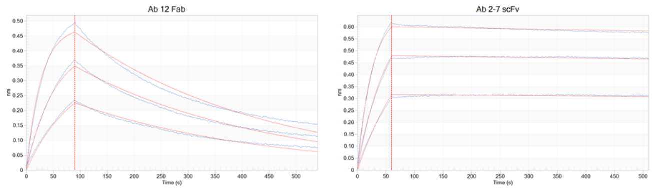

B

	K_D (M)	k_{on} (M^{-1} s^{-1})	k_{off} (s^{-1})	R^2
Ab 12 Fab	8.52E-09	3.39E+05	2.89E-03	0.9887
Ab 2-7 scFv	1.07E-10	4.52E+05	4.81E-05	0.9976

C

	Heavy Chain			Light Chain	
Ab	V-GENE and allele	J-GENE and allele	D-GENE and allele	V-GENE and allele	J-GENE and allele
Ab 12	HV4-59*11 F	HJ1*01 F	HD2-2*02 F	LV1-44*01 F	LJ1*01 F
Ab 2-7	HV2-5*01 F	HJ3*02 F	HD3-22*01 F	LV6-57*01 F	LJ3*02 F

D

```
                                FR1-IMGT              CDR1-IMGT    FR2-IMGT           CDR2-IMGT   FR3-IMGT                                   CDR3-IMGT        FW4-IMGT
MK540647 Homsap IGHV4-59*11 F   QVQLQESGP.GLVKPSETLSLTCTVS GGSI....SSHY WSWIRQPPGKGLEWIGY IYYS...GST NYNPSLK.SRVTISVDTSKNQFSLKLSSVTAADTAVYYC AR
Ab 12-HC                        EVQLVESGP.GVVSPSATLFLTCSVS GGSI....RTHS WNWIRQPPGKPLEWIGF IHHS...GAT NKNPSLK.SRVTISSDTSKNEFSLTLTSVTAADTAVYYC ARGPGILSY        WSRGTLVTVSS

Z73654 Homsap IGLV1-44*01 F     QSVLTQPPS.ASGTPGQRVTISCSGS SSNI....GSNT VNWYQQLPGTAPKLLIY SN.......N QRPSGVP.DRFSGSK..SGTSASLAISGLQSEDEADYYC AAWDDSLNG
Ab 12-LC                        LPVLTQPPS.ASGTPGQRVTISCSGS SSNI....GSNT VNWYQQLPGTAPKLLIY SN.......N QRPSGVP.DRFSGSK..SGTSASLAISGLQSEDEADYYC AAWDDSLNVHYV     FGSGTKVTVL
```

E

```
                                FR1-IMGT              CDR1-IMGT    FR2-IMGT           CDR2-IMGT   FR3-IMGT                                   CDR3-IMGT        FW4-IMGT
X62111 Homsap IGHV2-5*01 F      QITLKESGP.TLVKPTQTLTLTCTFS GFSLS..TSGVG VGWIRQPPGKALEWLAL IYWN...DDK RYSPSLK.SRLTITKDTSKNQVVLTMTNMDPVDTATYYC AHR
Ab 2-7-HC                       QVTLKESGP.TRVKPTQTLTLTCTFS GFSLS..TTGVG VGWIRQPPGKALEWLAL IYWN...DDK RYSPSLK.SRLTITKDTSKNQVVLTMTNMDPVDTATYYC ARISGSGYFYPFDI WGQGTTVTVSS

Z73673 Homsap IGLV6-57*01 F     NFMLTQPHS.VSESPGKTVTISCTRS SGSI....ASNY VQWYQQRPGSSPTTVIY ED.......N QRPSGVP.DRFSGSIDSSSNSASLTISGLKTEDEADYYC QSYDSSN
Ab 2-7-LC                       NFMLTQPHS.VSESPGKTVTISCTRS SGSI....ASNY VQWYQQRPGSSPTTVIY ED.......N QRPSGVP.DRFSGSIDSSSNSASLTISGLKAEDEADYYC QSYDSSSLWV       FGGGTKLTVL
```

Supplementary Figure 1: Characterization and analysis of anti-SARS-CoV-2 antibodies A) Biolayer interferometry traces for Ab 12 Fab (left) and 2-7 scFv (right). Abs were tested at 100, 50, and 25 nM against biotinylated RBD. The red trace shows the classical 1:1 binding model for the collected data (blue trace). **B)** Kinetic constants and R^2 values derived from the traces in **(A)** using a global fit of the indicated concentrations. **C)** Germline and allele assignments for Ab 12 and Ab 2-7**.** Sequences for the V region of **(D)** Ab 12 and **(E)** Ab 2-7 were aligned with their germline sequences with differences highlighted in red. Ab 12 HC is heavily mutated from the original IGHV4-59 sequences, whereas the LC shows minimal mutations outside of the FW1 region. Ab 2-7 has very few mutations compared to the germline sequences. Germline assignments and alignments were performed by IMGT V-QUEST[1,2].

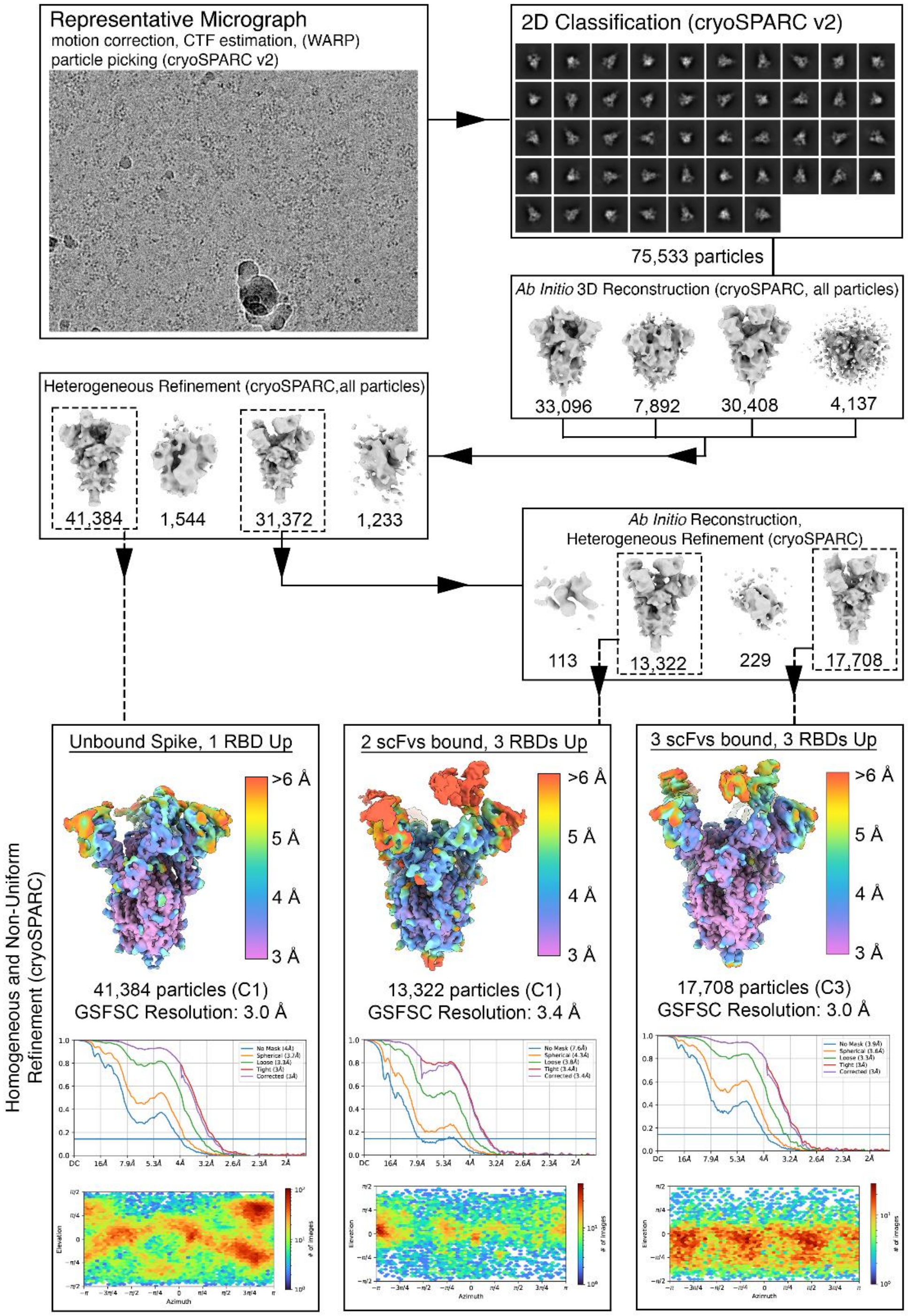

Supplementary Figure 2: Cryo-EM Processing Pipeline for SARS-CoV-2 S-D614G complexed with Ab 2-7 scFv

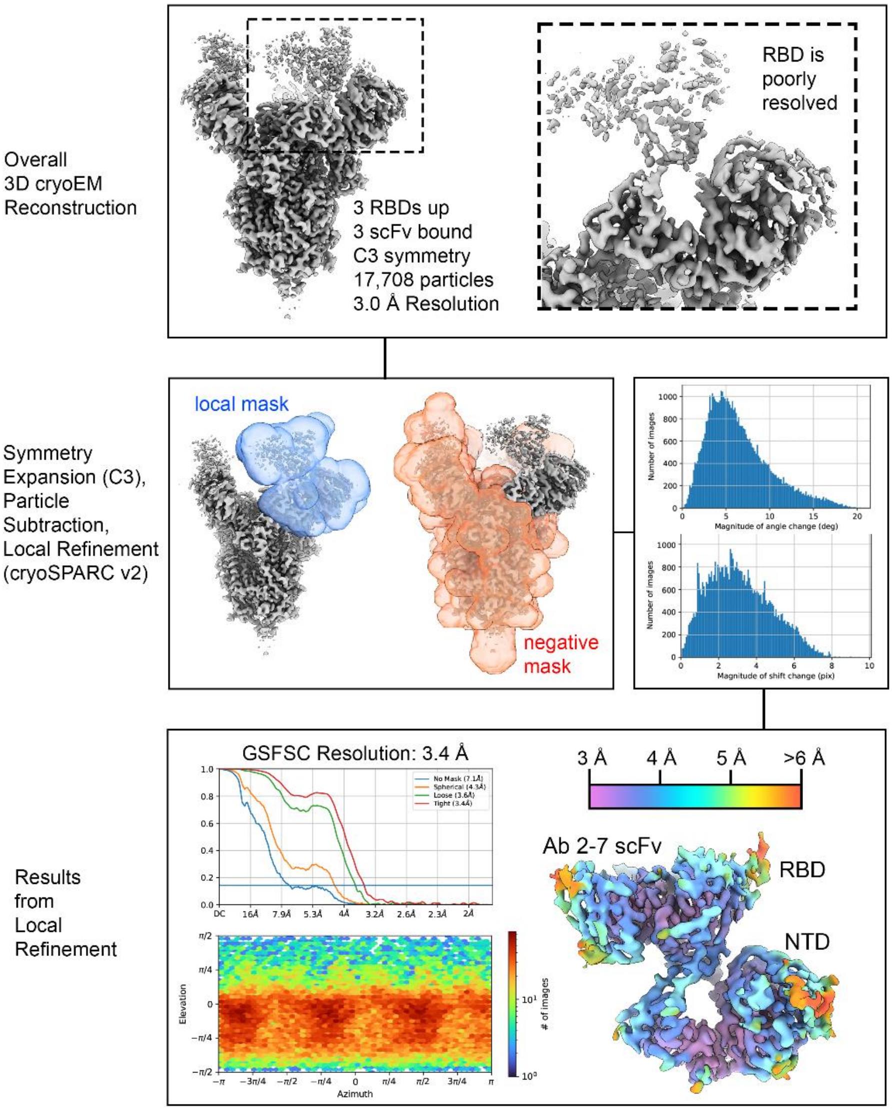

Supplementary Figure 3: Local Refinement of Ab 2-7 scFv bound to the spike RBD

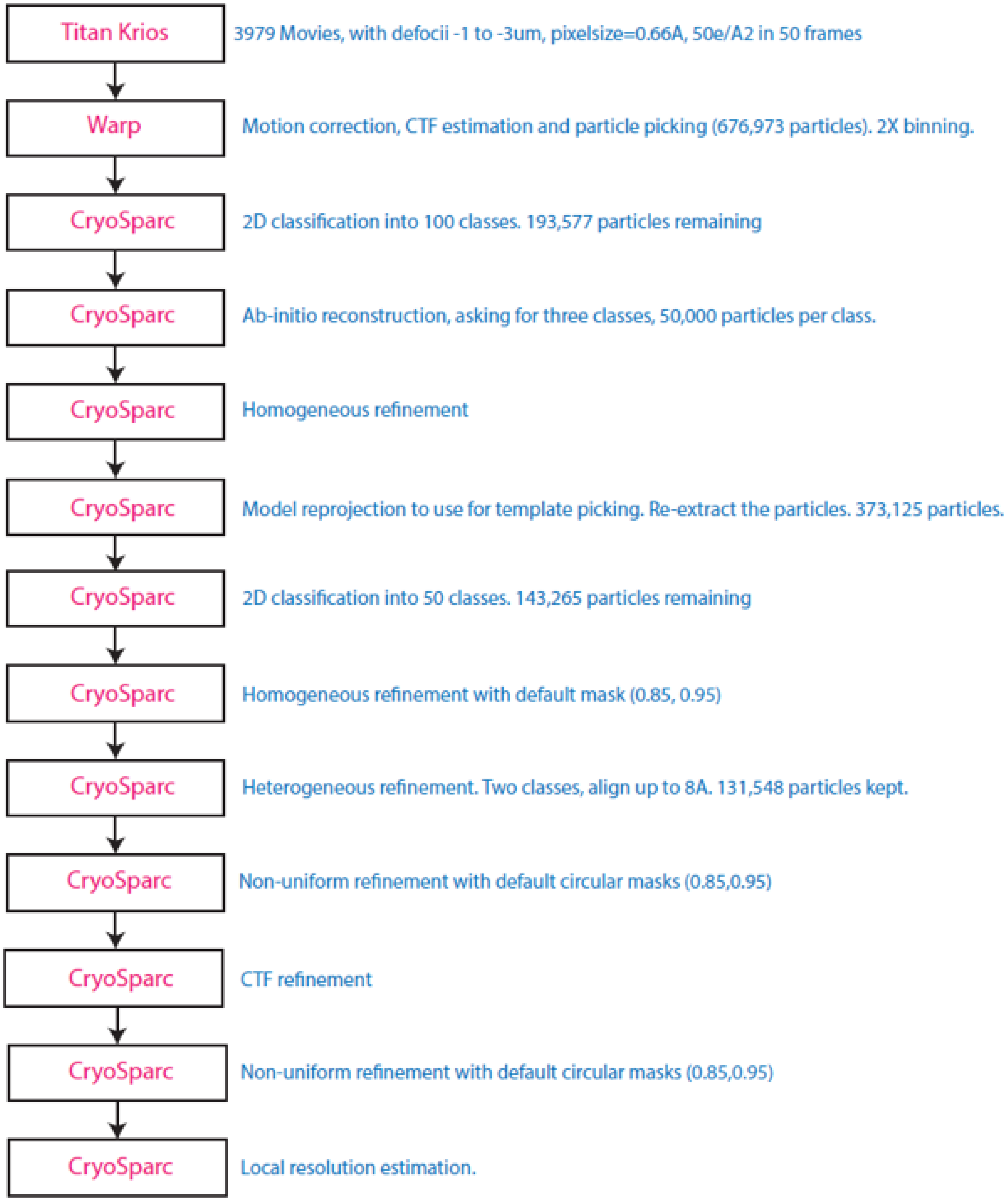

Supplementary Figure 4: Cryo-EM Processing Flowchart for SARS-CoV-2 S complexed with Ab 12 Fab

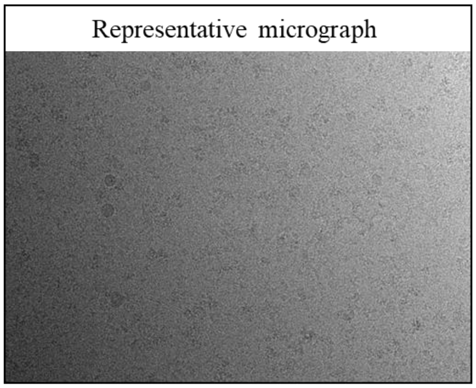
Representative micrograph

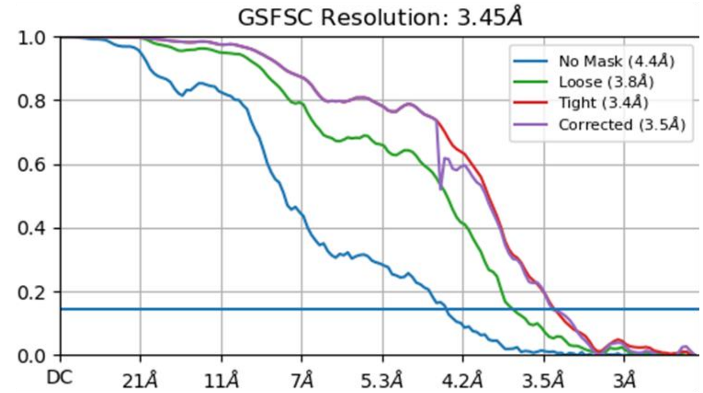
GSFSC Resolution: 3.45Å
1.0
0.8
0.6
0.4
0.2
0.0
DC
21Å
11Å
7Å
5.3Å
4.2Å
3.5Å
3Å
No Mask (4.4Å)
Loose (3.8Å)
Tight (3.4Å)
Corrected (3.5Å)

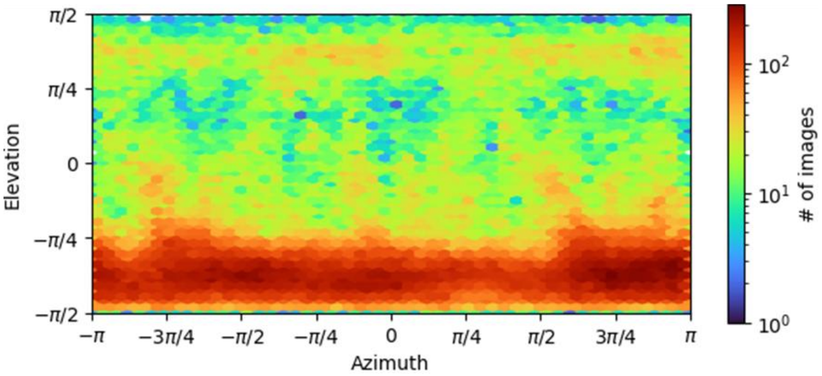
π/2
π/4
0
−π/4
−π/2
Elevation
−π
−3π/4
−π/2
−π/4
0
π/4
π/2
3π/4
π
Azimuth
10^2
10^1
10^0
# of images

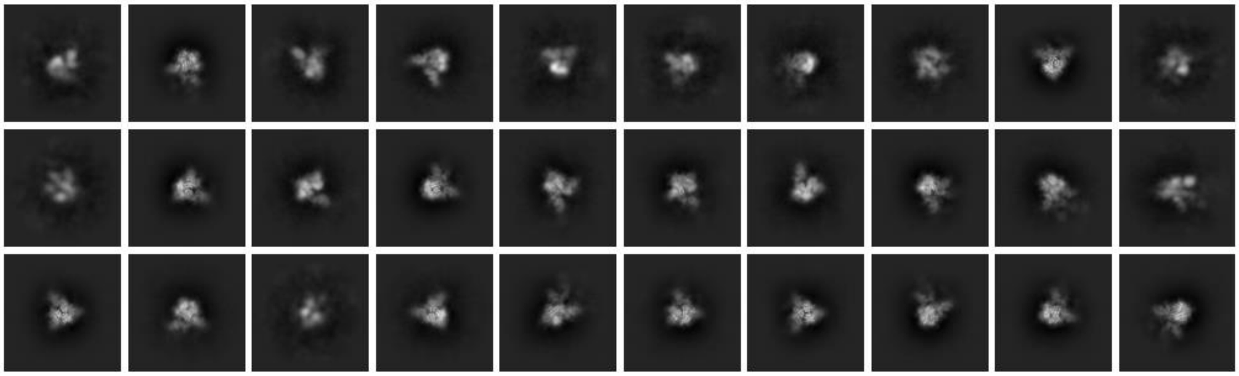

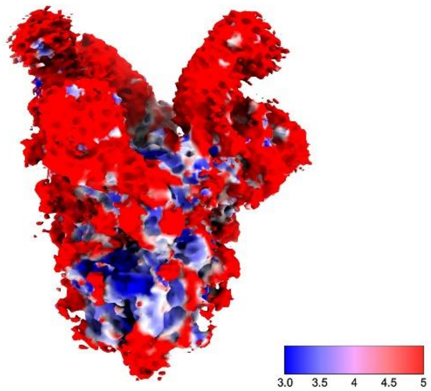

Supplementary Figure 5: Cryo-EM Processing for SARS-CoV-2 S-D614G complexed with Ab 12 Fab

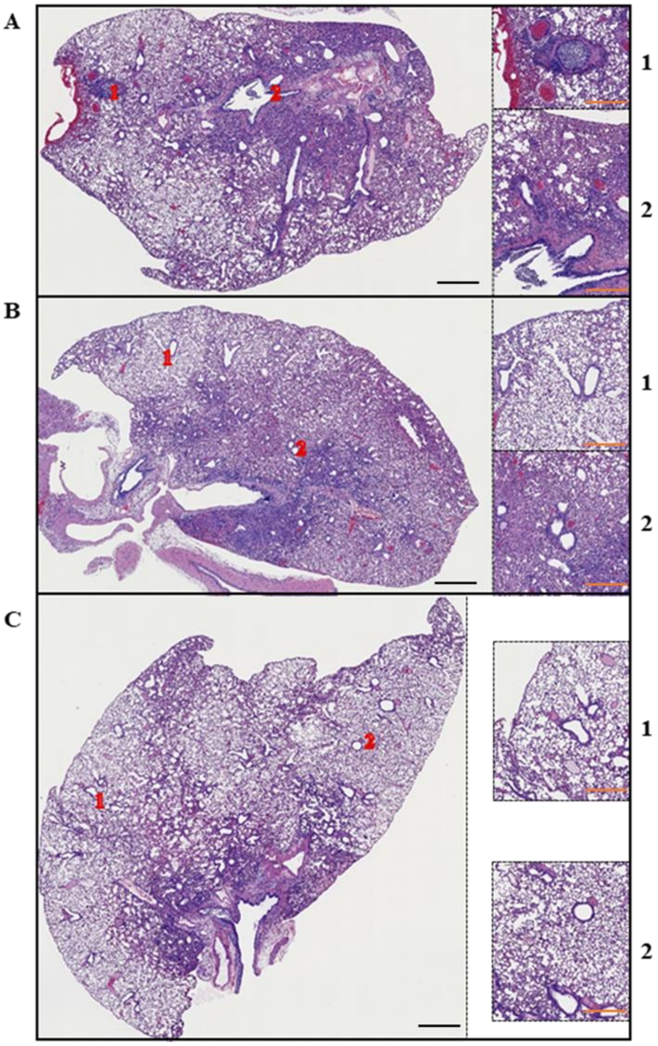

Supplementary Figure 6: Pathological analysis from lungs of Syrian golden hamsters treated prophylactically with Ab 12 or Ab 2-7 A) Representative image of stained control lung. Extensive consolidation with multiple foci of inflammatory infiltration. Magnified images (locations on low magnification images marked with numbers): (1) Airways are obstructed by inflammatory cells (combination of MNC and PMNs). (2) Airway epithelial hyperplasia notable. Perivascular cuffing and congestion prominent. **B)** Representative image of stained Ab 2-7 lung. Extensive consolidation with multiple foci of inflammatory infiltration. (1) Pleuritis noted, but less severe. (2) Fewer inflammatory cells in airways, but hyperplasia of airway epithelia is still prominent. **C)** Representative image of stained Ab 12 lung. Consolidation markedly reduced, with fewer and smaller foci of inflammatory infiltration. Infiltrating inflammatory cells present in some airways. (1) Pleuritis is moderate relative to control. (2) Airway epithelial hypertrophy still present. Scale bars are 1 mm in the main (left) panels and 0.5 mm in the zoomed images (right). One slide was prepared for each animal (n=5) and a representative slide was chosen for each group.

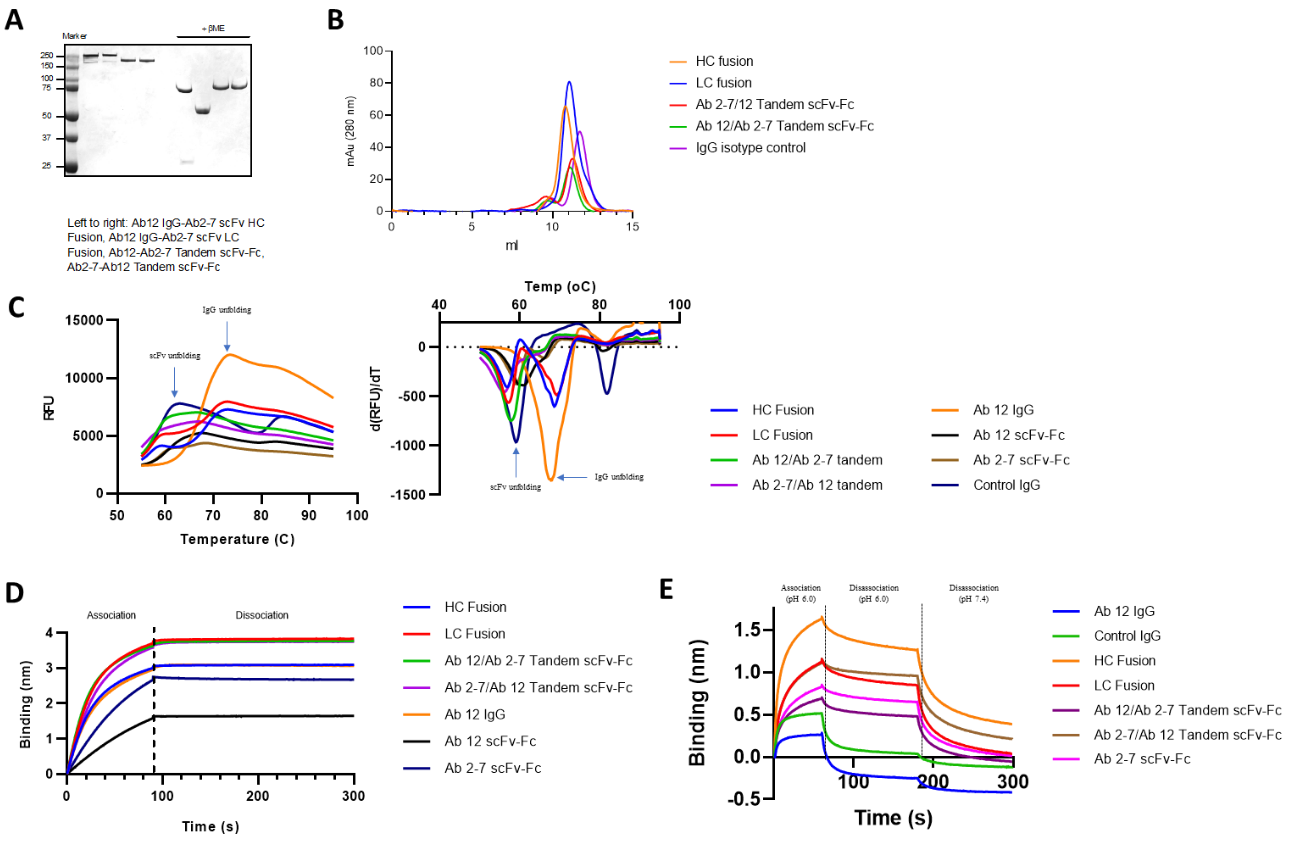

Supplementary Figure 7: Biochemical characterization of bispecific antibodies. A) SDS-PAGE gel of IgG fusions and tandem scFv-Fcs. The first four lanes are non-reduced samples and the last 4 lanes are reduced with 10% BME **B)** Size exclusion chromatography of the bispecific antibodies showing peaks at the expected elution volume and minimal aggregation **C)** Thermal stability of our mono and bispecific constructs measured by SYPRO Orange thermal shift assay. Graph on the left is the raw fluorescence vs temperature, graph on the right is the change of fluorescence vs temp. Melting peaks of composite antibodies is similar to that of the individual components, suggesting that the fusions do not significantly affect the stability of the parental IgG or scFv **D)** BLI curves for bispecific and monospecific Abs show strong binding the RBD. Ab 12 is the dominant binding moiety in these bispecifics and that is also shown in the binding curves here. **E)** Engineered BsAbs display similar kinetics to FcRn as parental antibodies, binding at pH 6 and disassociating at pH 7.4. Samples were tested with n=1 with the exception of (C) which was performed as 3 biologically independent samples with the mean values presented.

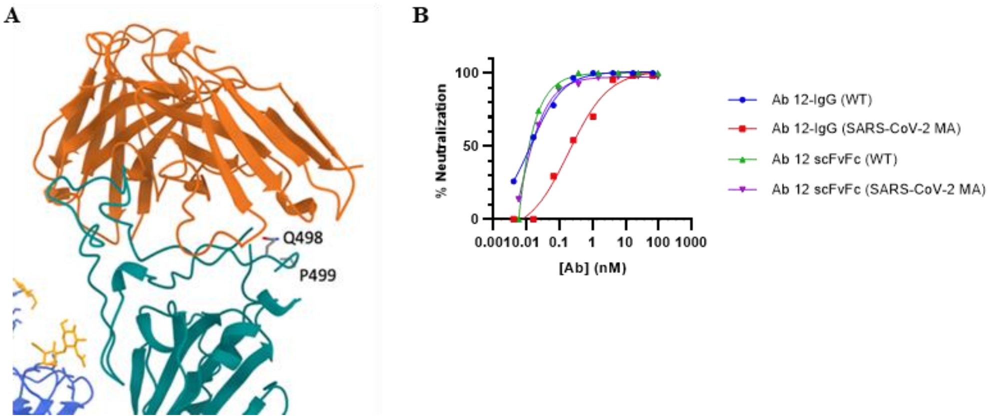

Supplementary Figure 8: *In vitro* neutralization of a mouse adapted strain of SARS-CoV-2 A) Ribbon model showing Ab 12 (orange) bound to the RBD (teal), with the mutations in the mouse adapted virus labeled. **B)** Mutations in the mouse adapted strain do not affect Ab 12 scFv-Fc neutralization, however the IgG format exhibits a decrease in effectiveness. Mean values are presented with n=2 biologically independent samples. Source data are provided as a Source Data file.

A

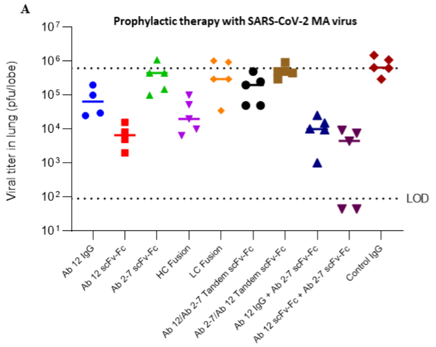

	Mean PFU/lobe	Fold improvement
Ab 12 IgG	8.88E+04	9.41
Ab 12 scFv-Fc	7.81E+03	106.88
Ab 2-7 scFv-Fc	4.50E+05	1.86
HC Fusion	3.78E+04	22.09
LC Fusion	5.23E+05	1.6
Ab 12/Ab 2-7 Tandem scFv-Fc	2.10E+05	3.98
Ab 2-7/Ab 12 Tandem scFv-Fc	5.35E+05	1.56
Ab 12 scFv-Fc + Ab 2-7 scFv-Fc	4.37E+03	191.16
Ab 12 IgG + Ab 2-7 scFv-Fc	1.21E+04	69.01
αPD1 IgG	8.35E+05	1

B

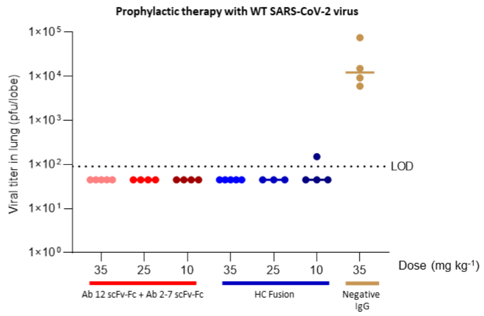

Supplementary Figure 9. Prophylactic efficacy against SARS-CoV-2 virus in mouse models A) Prophylactic efficacy of Ab 12 and Ab 2-7 mono- and bi- specific antibodies were tested in aged Balb/c mice. Mean PFU after infection is tabulated in the table to the right of the chart, with fold improvement relative the αPD1 negative control. **B)** BsAb-HC fusion and scFv-Fc mixture were selected for prophylactic testing in transgenic hACE2 mice with WT SARS-CoV-2 virus. Both treatments lead to reduction of viral titers below the limit of detection in all samples except for one animal treated with 10 mg kg^{-1} of BsAb-HC that showed residual virus. Data from individual mice with mean values are presented on both plots. Source data are provided as a Source Data file.

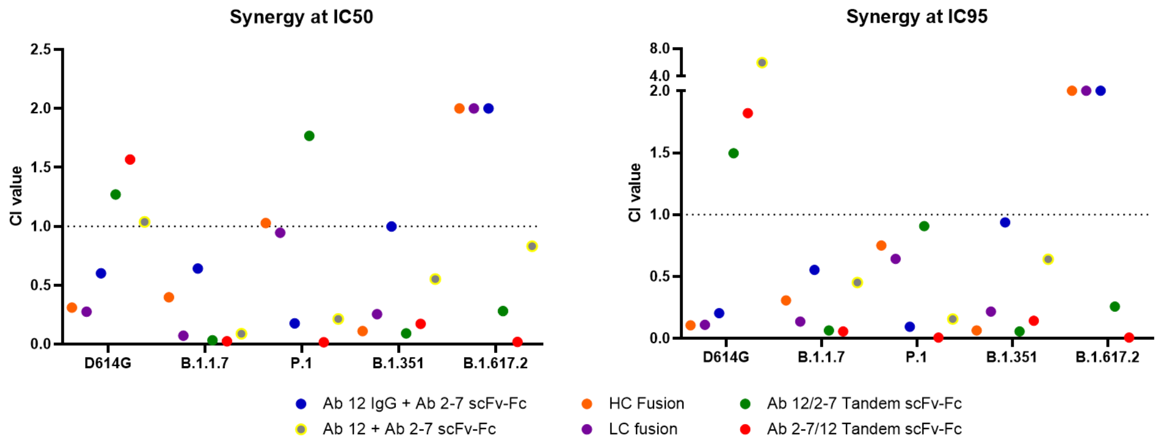

Supplementary Figure 10. Combination Index (CI) values were calculated for the IC50 and IC95 values using in vitro neutralization curves and CompuSyn. CI values are used to identify synergy by determining if the effect seen by a combination of two therapies is greater than the expected sum of their therapies. CI < 1 indicates synergy, C = 1 indicates an additive effect, and CI > 1 indicates an antagonistic effect where the result of the combination therapy is less than what would be expected when the two components are simply added together. Against the majority of variants tested, the BsAb therapies display potent synergy, though a few instances of additive and antagonistic effects are seen. Source data are provided as a Source Data file.

EM data collection for Ab 2-7

Microscope	FEI Titan Krios
Voltage (kV)	300
Detector	Gatan K3
Calibrated magnification (Å/pix)	0.66
Exposure rate (e^-/pix/sec)	8
Frames per exposure	80
Exposure (e^-/Å^2)	80
Defocus range (mm)	1.5-2.5
Tilt angle (˚)	30
Micrographs collected	4,851
Micrographs used	4,226
Particles extracted (total)	1,294,509
Automation software	SerialEM
Sample	SARS-CoV-2 S D614G plus Ab 2-7 scFv

3D reconstruction statistics

EMDB IDs global:	EMD-25689	EMD-25690	EMD-25711
composite:	EMD-25663		
Complex Composition	Spike complexed with Ab 2-7 scFv	RBD:scFv subcomplex	Unbound Spike
Particles	17,708	53,124	41,384
Symmetry	C3	C1	C1
Map sharpening B-factor	118.5	141.5	117.1
Unmasked resolution at 0.5 FSC (Å)	8.1	10.8	8.5
Masked resolution at 0.5 FSC (Å)	3.4	3.9	3.5
Unmasked resolution at 0.143 FSC (Å)	3.9	7.0	4.0
Masked resolution at 0.143 FSC (Å)	3.0	3.4	3.0

Local resolution range at 0.5 FSC (Å):			
minimum	2.5	2.8	2.6
25th percentile	4.1	3.5	4.0
Median	6.0	4.8	6.1
75th percentile	6.9	6.0	7.2
Maximum	46.9	51.7	43.7

Model refinement & validation statistics

PDB IDs	7T3M	7T67
Refinement package	Phenix	Phenix
Refinement tool(s)	Real-space refinement; combine_focused_maps	Real-space refinement
Refinement strategies	min global; local_grid_search; adp; ss, rotamer and Ramachandran restraints	min global; local_grid_search; adp; ss, rotamer and Ramachandran restraints
Composition		
Amino acids	3771	3,075
RMSD bonds (Å)	0.002	0.003
RMSD angles (º)	0.56	0.73
Average B-factors		
Amino acids	51.3	134.76
Ramachandran		
Favored (%)	97.06	97.19
Allowed (%)	2.94	2.81
Outliers (%)	0	0
Rotamer outliers (%)	0.03	0
C-beta outliers (%)	0	0
CaBLAM outliers (%)	1.64	2.69
CC (mask)	0.82	0.80

MolProbity score	1.32	1.44
Clash score	3.62	5.49
EMRinger score	3.1	2.3

Supplementary Table 1: Cryo-EM of S-D614G complexed with Ab 2-7 scFv: Data collection, refinement statistics and model validation.

	Scores→	0	1	2	3	4
**A**	Extent of inflammation (% tissue involved)	0	<10	10-30	30-60	>60
**B**	Inflammatory foci type	No inflammation	Patchy inflammatory foci, few (<2)	Patchy inflammatory foci, many (>2)	Large inflammatory foci, few (<2)	Large inflammatory foci, many (>2)
**C**	Inter-alveolar septa (IAS)	Thin and delicate	Thickened in <10% HPF	Thickened in <30% HPF	Thickened in <60% HPF	Thickened in >60% HPF
**D**	Air ways	Clear; no cells	Few cells in air way	Moderate cells in air way	More cells in air way; Epithelial hyperplasia	Occlusion of air way/epithelial hyperplasia or desquamation
**E**	Alveoli/ perivascular cuff/blood vessels/ pleuritis/cell types	Clear; no inflammatory cells	Few cells/ Few PMN or MNC	Moderate cells/ PVC/ mild congestion/mild pleuritis/mostly MNC	More cells/PVC/ more congestion and pleuritis/ more MNC and PMN	Abundant cells/large PVC/severe congestion or pleuritis/mixed cells
	*Adapted from Matute-Bello et al. (2011).					

Supplementary Table 2: Criteria for lung histopathology scoring
HPF – high power field (>10x); PMN – polymorphonuclear cells/heterophils; MNC – mononuclear cells including lymphocytes and macrophages); PVC – Peri-vascular cuff.

IC50 (nM)	Ab 12 IgG	Ab 12 scFv-Fc	Ab 2-7 scFv-Fc	HC Fusion	LC Fusion	Ab 12/2-7 Tandem scFv-Fc	Ab 2-7/12 Tandem scFv-Fc	Ab 12 IgG+Ab2-7 scFv-Fc	Ab12 scFv-Fc +Ab 2-7 scFv-Fc	Control IgG
**D614G**	14.11	15.72	N/A	2.221	5.082	3.516	3.212	9.327	4.392	N/A
**B.1.1.7**	4.325	1.65	1497	1.85	0.7511	0.6292	0.6337	3.472	1.073	N/A
**B.1.351**	178.4	3809	130.4	12.58	12.67	51.8	6.837	67.5	91.57	N/A
**P.1**	18.24	62.88	6.702	2.896	2.781	8.069	0.2712	1.903	1.023	N/A
**B.1.617.2**	N/A	N/A	102.1	132.2	439.1	158.6	9.587	133.6	115.1	N/A

Supplementary Table 3: IC50 for mono and BsAb neutralization of VOC. IC50 values from *in vitro* variant neutralization in **Fig. 7** were calculated using a sigmoidal 4PL interpolation in GraphPad Prism. Abs that did not achieve at least 10% neutralization at the highest concentration or with IC50 values >10 fold greater than the highest tested concentration were changed to N/A.

Supplementary References:

1. Brochet, X., Lefranc, M. P. & Giudicelli, V. IMGT/V-QUEST: the highly customized and integrated system for IG and TR standardized V-J and V-D-J sequence analysis. *Nucleic Acids Res.* **36**, 503–508 (2008).
2. Lefranc, M. P. *et al.* IMGT unique numbering for immunoglobulin and T cell receptor variable domains and Ig superfamily V-like domains. *Dev. Comp. Immunol.* **27**, 55–77 (2003).
3. Hodcroft, E. B. CoVariants: SARS-CoV-2 Mutations and Variants of Interest. https://covariants.org/ (2021).
