# Supplementary material for: IgG-like bispecific antibodies with potent and synergistic neutralization against circulating SARS-CoV-2 variants of concern
Source: Nat Commun. 2022 Oct 3;13:5814. doi: 10.1038/s41467-022-33030-4 (PMC9528872; doi:10.1038/s41467-022-33030-4)
Supplement: Supplementary file 2 — Reporting Summary [file 41467_2022_33030_MOESM2_ESM.pdf]

nature portfolio

Corresponding author(s): Wayne Marasco

Last updated by author(s): Aug 3, 2022

Reporting Summary

Nature Portfolio wishes to improve the reproducibility of the work that we publish. This form provides structure for consistency and transparency in reporting. For further information on Nature Portfolio policies, see our Editorial Policies and the Editorial Policy Checklist.

Statistics

For all statistical analyses, confirm that the following items are present in the figure legend, table legend, main text, or Methods section.

n/a	Confirmed	
☐	☒	The exact sample size (*n*) for each experimental group/condition, given as a discrete number and unit of measurement
☐	☒	A statement on whether measurements were taken from distinct samples or whether the same sample was measured repeatedly
☐	☒	The statistical test(s) used AND whether they are one- or two-sided<br>*Only common tests should be described solely by name; describe more complex techniques in the Methods section.*
☒	☐	A description of all covariates tested
☐	☒	A description of any assumptions or corrections, such as tests of normality and adjustment for multiple comparisons
☐	☒	A full description of the statistical parameters including central tendency (e.g. means) or other basic estimates (e.g. regression coefficient) AND variation (e.g. standard deviation) or associated estimates of uncertainty (e.g. confidence intervals)
☐	☒	For null hypothesis testing, the test statistic (e.g. *F*, *t*, *r*) with confidence intervals, effect sizes, degrees of freedom and *P* value noted<br>*Give P values as exact values whenever suitable.*
☒	☐	For Bayesian analysis, information on the choice of priors and Markov chain Monte Carlo settings
☒	☐	For hierarchical and complex designs, identification of the appropriate level for tests and full reporting of outcomes
☒	☐	Estimates of effect sizes (e.g. Cohen's *d*, Pearson's *r*), indicating how they were calculated

Our web collection on statistics for biologists contains articles on many of the points above.

Software and code

Policy information about availability of computer code

Data collection	Sartorius' Octet Data Acquisition software V10.0.0.87, BD's FACSDiva software, serialEM
Data analysis	Prism (V 6.05, 8.4.2, 9); Sartorius' Octet Data Analysis software V10; FlowJo V10; cryoSPARC v2 and v3

For manuscripts utilizing custom algorithms or software that are central to the research but not yet described in published literature, software must be made available to editors and reviewers. We strongly encourage code deposition in a community repository (e.g. GitHub). See the Nature Portfolio guidelines for submitting code & software for further information.

Data

Policy information about availability of data

All manuscripts must include a data availability statement. This statement should provide the following information, where applicable:

- Accession codes, unique identifiers, or web links for publicly available datasets
- A description of any restrictions on data availability
- For clinical datasets or third party data, please ensure that the statement adheres to our policy

The datasets generated during and analyzed during the current study are included in the Source Data file. Atomic models generated in this study have been deposited into the PDB with accession number 7T3M (spike with 3 Ab 2-7 scFvs) and 7T67 (apo spike trimer). The corresponding cryo-EM density maps generated in this study have been deposited into the Electron Microscopy Data Bank with accession numbers EMD-25689 and EMD-25663 (Spike complexed with Ab 2-7 scFv), EMD-25690 (RBD:scFv subcomplex), EMD-25711 (unbound spike), EMD-25618 (Spike complexed with Ab 12).

Field-specific reporting

Please select the one below that is the best fit for your research. If you are not sure, read the appropriate sections before making your selection.

☒ Life sciences ☐ Behavioural & social sciences ☐ Ecological, evolutionary & environmental sciences

For a reference copy of the document with all sections, see nature.com/documents/nr-reporting-summary-flat.pdf

Life sciences study design

All studies must disclose on these points even when the disclosure is negative.

Sample size	For experiments conducted in the Baric Lab, sample sizes were chosen based on previous experience with live SARS-CoV-2 neutralization assays and mouse models of coronavirus infection. For hamster studies, the number of samples was the minimum number required to obtain scientifically valid results. Five animals per experimental group were used, based on 100% mortality following infection with filoviruses, which allowed statistical assessment at the 95% confidence level (1-tailed Fisher exact test). For CryoEM, sample size (number of particles) included in the final reconstructions was determined according to standard practices in the single-particle cryoEM field. Specifically, cryoSPARC sorts particles of similar conformation into 2D and 3D classes, which are then refined according to the gold-standard FSC criterion.
Data exclusions	Micrographs with a poor fit (x>7A) to the contrast transfer function were excluded from particle picking. Picked particles that did not sort into high quality 2D or 3D classes were excluded. Data from other experiments was not excluded
Replication	Experiments were conducted in two different mouse models. In vitro neutralization experiments in the Baric Lab were conducted in duplicate. All attempts at replication of hamster models in the stated conditions were successful. FACS experiments are performed in duplicate, kinetic/binding measurements in the Marasco lab are performed in triplicate. High resolution cryoEM structure determination was not reproduced.
Randomization	Animals were assigned to experimental groups randomly. Age and sex-matched control animals were used.
Blinding	Investigators were not blinded for mice experiments or cryoEM studies. Blinding in cryoEM studies would preclude the ability to analyze data, since the person(s) building the model must know the identity its contents. UTMB Animal Resource Center veterinary staff was blinded for the antibodies' administration to experimental hamster groups.

Reporting for specific materials, systems and methods

We require information from authors about some types of materials, experimental systems and methods used in many studies. Here, indicate whether each material, system or method listed is relevant to your study. If you are not sure if a list item applies to your research, read the appropriate section before selecting a response.

Materials & experimental systems

n/a	Involved in the study	
☐	☒	Antibodies
☐	☒	Eukaryotic cell lines
☒	☐	Palaeontology and archaeology
☐	☒	Animals and other organisms
☒	☐	Human research participants
☒	☐	Clinical data
☒	☐	Dual use research of concern

Methods

n/a	Involved in the study	
☒	☐	ChIP-seq
☐	☒	Flow cytometry
☒	☐	MRI-based neuroimaging

Antibodies

Antibodies used	anti-SARS-CoV Spike Antibody, Rabbit PAb, Antigen Affinity Purified (Sino Biological cat #40150-T62-COV2); HRP Donkey anti-rabbit IgG (minimal x-reactivity) Antibody (Biolegend cat# 406401); Goat anti-Human IgG-Fc Fragment Antibody HRP Conjugated (Bethyl cat# A80-104P); APC anti-human IgG-Fc (Biolegend cat# 409306); anti-SARS-CoV-2 spike mono- and bi-specific antibodies produced by the Marasco Lab and identified in this manuscript, monospecific antibodies were also expressed by the McLellan and Saphire labs for CryoEM; CR3022 genes synthesized from published sequences and expressed in the Marasco Lab; AM130 and AM122 were purchased from Acro Biosystems.
Validation	anti-SARS-CoV-2 mono- and bi-specific antibodies were tested for binding to irrelevant cancer related proteins to ensure specificity. 40150-T62-COV2 is validated by Sino Biological to cross react with SARS-CoV-2 S1 and RBD in both Western blot and ELISA. Expression plasmids encoding antibodies identified in this study were confirmed via Sanger sequencing and expressed monoclonals were confirmed via CryoEM

Eukaryotic cell lines

Policy information about cell lines

Cell line source(s)	The Baric lab used Vero E6 cells obtained from USAMRIID in 2003. The Griffiths Lab used cells from BEI Resources, catalog NR-596 [VERO C1008 (E6), Kidney (African green monkey) working cell bank]. The Bukreyev Lab used Vero E6, ATCC cat # CRL-1586. 293T/17 and Expi293F cells used in the Marasco Lab were acquired from ATCC (CRL-11268) and Thermo Fisher (A14635) respectively. McLellan Lab used Hek293F from Thermo Fisher Scientific.
Authentication	Cell lines were not authenticated except by vendor/distributor
Mycoplasma contamination	Cells used in the Baric and Marasco Labs were were not tested for mycoplasma. Cells used by the Griffiths Lab were tested via Hoechst DNA stain, Agar & broth culture, or DNA detection by PCR and no mycoplasma was detected. Cells in the Bukreyev Lab also tested negative for mycoplasma contamination. Cells are routinely tested for mycoplasma contamination in the McLellan Lab (every three to six months) and were negative during the course of this study.
Commonly misidentified lines (See ICLAC register)	Vero E6 cells were used for viral infection; HEK293F cell line was used because it is designed for expression of recombinant protein and the structural portion of this study depends only on the quality of the purified recombinant protein.

Animals and other organisms

Policy information about studies involving animals; ARRIVE guidelines recommended for reporting animal research

Laboratory animals	1-year old female BALB/c mice were obtained from Envigo. hACE2 transgenic mice were derived in our lab and maintained at UNC at Chapel Hill. Female golden Syrian hamsters, aged 6–7 weeks maintained at UTMB.
Wild animals	This study did not involve wild animals
Field-collected samples	This study did not involve field-collected samples
Ethics oversight	Recombinant viruses were approved by UNC Chapel Hill IBC under Schedule G 73790. Mice work was approved by UNC Chapel Hill IACUC under protocol 19-168. Golden hamster work was approved under animal protocol # 2004049 by the Institutional Animal Care and Use Committee (IACUC) of the University of Texas Medical Branch at Galveston (UTMB).

Note that full information on the approval of the study protocol must also be provided in the manuscript.

Flow Cytometry

Plots

Confirm that:

- ☐ The axis labels state the marker and fluorochrome used (e.g. CD4-FITC).
- ☐ The axis scales are clearly visible. Include numbers along axes only for bottom left plot of group (a 'group' is an analysis of identical markers).
- ☐ All plots are contour plots with outliers or pseudocolor plots.
- ☐ A numerical value for number of cells or percentage (with statistics) is provided.

Methodology

Sample preparation	FACS was performed using 293T cells transduced to express membrane bound SARS-CoV-2 spike. Prior to spike shedding experiment, transduced cells were sorted to decrease the number of non-transduced cells.
Instrument	BD FACSCanto II Cytometer 4/2/2 Sys IVD
Software	Samples were collected using BD FACSDiva and analyzed in FlowJo.
Cell population abundance	10,000 events were collected for each sample and >85% expressed the SARS-CoV-2 spike as determined by BFP expression (cells were transduced with SARS-CoV-2 spike-IRES-BFP)
Gating strategy	After FSC/SSC gating, BFP+ cells were selected. From there, antibody binding to the cells was measured.

☐ Tick this box to confirm that a figure exemplifying the gating strategy is provided in the Supplementary Information.
